# Efficacy of nebulized salbutamol in transient tachypnea of the newborn: A systematic review and meta-analysis

**DOI:** 10.1371/journal.pone.0359663

**Published:** 2026-10-01

**Authors:** Nanthida Phattraprayoon, Nut Koonrungsesomboon, Mingkwan Na Takuathung

**Affiliations:** 1 Princess Srisavangavadhana Faculty of Medicine, Chulabhorn Royal Academy, Bangkok, Thailand; 2 Department of Pharmacology, Faculty of Medicine, Chiang Mai University, Chiang Mai, Thailand; 3 Clinical Research Center for Food and Herbal Product Trials and Development (CR-FAH), Faculty of Medicine, Chiang Mai University, Chiang Mai, Thailand; Hacettepe University, Faculty of Medicine, TÜRKIYE

## Abstract

Transient tachypnea of the newborn (TTN) commonly affects near- and full-term infants and often requires respiratory support. The use of nebulized salbutamol to improve respiratory outcomes has been of interest, though its clinical efficacy remains controversial. This systematic review and meta-analysis aimed to evaluate whether nebulized salbutamol improves clinical outcomes in affected infants. We searched PubMed, Scopus, Embase, and the Cochrane Library for randomized controlled trials (RCTs) evaluating the efficacy of nebulized salbutamol in infants with TTN. We used a random-effects model to calculate mean differences (MDs) with 95% confidence intervals (CIs). Twenty-five studies were included in the systematic review, comprising seventeen RCTs for meta-analysis involving 1,317 infants. The analysis showed that salbutamol significantly decreased the duration of respiratory support (MD: −14.85 hours; 95% CI [−25.05, −4.65]; p = 0.004), duration of continuous positive airway pressure (MD: −18.19 hours; 95% CI [−30.03, −6.35]; p = 0.003), duration of oxygen requirement (MD: −24.57 hours; 95% CI [−35.03, −14.10]; p < 0.001), and length of hospitalization (MD: −1.17 days; 95% CI [−1.69, −0.65]; p < 0.001). However, there was no significant decrease in duration of tachypnea (MD: −9.93 hours; 95% CI [−25.42, 5.56]; p = 0.21) or duration of neonatal intensive care unit stay (MD: −0.84 days; 95% CI [−2.62, 0.94]; p = 0.36). Subgroup analysis for respiratory support duration showed no significant effect in infants with clinical score of TTN < 5 (MD: −10.88 hours; 95% CI [−24.48, 2.73]; p = 0.12), while the certainty of evidence for all outcomes was low to very low. Nebulized salbutamol may improve clinical outcomes relevant to the evolution of the disease in TTN infants, with potential benefits in certain subgroups such as those with greater disease severity, though the available evidence is of low to very low certainty. Therefore, our confidence in its efficacy in treating TTN remains limited.

## Introduction

Transient tachypnea of the newborn (TTN) is a respiratory condition characterized by respiratory distress, including tachypnea (>60 breaths/min), nasal flaring, grunting, and retractions. These symptoms typically manifest shortly after birth [1]. Approximately 10% of infants born at 33–34 weeks are affected, 5% at 35–36 weeks, and under 1% among those born at 37 weeks or more of gestational age [1,2]. Affected infants may require respiratory support, including oxygen supplementation and continuous positive airway pressure (CPAP).

The pathophysiology of TTN involves impaired clearance of lung fluid in newborns. During fetal development, lung fluid secretion occurs through active chloride ion transport, with sodium ions and water passively flowing into the alveolar spaces [3]. Toward the end of pregnancy, hormonal surges, particularly in glucocorticoids and thyroid hormones, activate sodium absorption channels. Fetal epinephrine released during labor stimulates β_2_ adrenergic receptors, which further activates epithelial sodium channels (ENaCs) and promotes lung fluid clearance.

Sodium entering through epithelial sodium channels is then extruded from the cell by basolateral sodium potassium ATPase, whose capacity is rapidly increased by the postnatal rise in oxygen, completing the transport of sodium and consequently water out of the airspace into the interstitial tissue [4]. Insufficient labor stress or inadequate hormonal signaling can impair this sodium driven fluid absorption mechanism, resulting in retained lung fluid [5].

In an animal study using normal rodent models, Mutlu et al. [6] demonstrated that cystic fibrosis transmembrane conductance regulator upregulation can accelerate alveolar fluid clearance in a beta-adrenergic receptor dependent manner, demonstrating a functional interdependency between cystic fibrosis transmembrane conductance regulator and beta-adrenergic receptor signaling in active sodium transport. Whether this specific mechanism operates similarly during the neonatal lung fluid transition relevant to transient tachypnea of the newborn has not been directly established.

Medications such as salbutamol, a β_2_-adrenergic receptor agonist, stimulate alveolar ENaCs and may enhance sodium transport and promote fluid absorption from the alveoli [7]. Other pharmacological and supportive interventions, including epinephrine, budesonide, diuretics, and fluid restriction, have also been studied for TTN [8], though these act through distinct or less well-established mechanisms and are supported by considerably more limited evidence. These treatments have not yet been adopted as standard therapeutic practice.

Several recent studies have explored whether salbutamol administration can accelerate lung fluid clearance and improve clinical outcomes in infants with TTN [9,10]. Therefore, in this systematic review and meta-analysis, we aimed to evaluate the efficacy of salbutamol in improving clinical outcomes among infants with TTN.

## Materials and methods

### Literature search and data collection

A comprehensive systematic literature search was performed using the PubMed, Scopus, Embase, and Cochrane Library databases to identify relevant studies published before April 26, 2026. The search strategy incorporated the following terms: (Transient tachypnea OR Transient tachypnoea OR TTN OR TTNB) AND (infant OR neonate OR preterm OR term). The reference lists of the identified articles were manually searched to identify additional relevant publications. The systematic review and meta-analysis were conducted in accordance with the Preferred Reporting Items for Systematic Reviews and Meta-Analyses (PRISMA) 2020 guidelines. The study protocol was prospectively registered with PROSPERO (registration number: CRD42025644147). An exemption certificate was obtained from the Research Ethics Committee of Princess Srisavangavadhana Faculty of Medicine, Chulabhorn Royal Academy (EC 039/2568).

### Eligibility criteria

Randomized controlled trials (RCTs) that compared nebulized salbutamol with a placebo and/or standard of care (control group) were included, without language restrictions. Only human studies involving preterm and term neonates with TTN were eligible for inclusion.

### Data extraction and quality assessment

Two researchers (NP and MN) independently assessed the relevance, study design, methodology, and outcomes of each study using the inclusion criteria. Any discrepancies were resolved through discussion or, if needed, arbitration by a third researcher (NK).

The efficacy of the treatment was evaluated using the following respiratory outcomes: duration of tachypnea (hours), duration of respiratory support (hours), duration of CPAP use (hours), and duration of oxygen requirement (hours). The time to initiate feeding (hours), duration of hospitalization and neonatal intensive care unit (NICU) stay (days) were also assessed. In addition, we categorized TTN severity using clinical scores as defined in the original studies, which employed three different scoring systems. The first was the Respiratory Distress Assessment Score, which is used to evaluate expiratory grunting, supraclavicular retraction, subcostal retraction, cyanosis, and nasal flaring. The second was Downes’ score, which is used to assess the respiratory rate, cyanosis, retractions, grunting, and air entry. The third was the Silverman–Anderson Retraction Score, which is used to assess chest movement, intercostal retraction, xiphoid retraction, nasal flaring, and expiratory grunting. Using these clinical scoring systems, patients were subgrouped according to their mean clinical score for TTN severity (hereafter, “TTN clinical score”), classified as <5 or ≥5, consistent with the original studies [11].

Regarding unclear or missing data, corresponding authors of the included studies were contacted via email for clarification. The risk of bias was assessed using the revised Cochrane risk-of-bias tool for randomized trials (RoB 2) [12], and the studies were categorized as low risk, high risk, or having some concerns. The findings were visually summarized using the robvis tool.

### Data synthesis and statistical analysis

Meta-analyses were conducted using RevMan software. Statistical significance was set at p < 0.05. Weighted mean differences (MDs) for all continuous outcomes were calculated with 95% confidence intervals (CIs) using a random-effects model. When studies reported data as median with interquartile range (IQR), established statistical conversion formulas were applied to transform these values into mean and standard deviation (SD) for analysis [13,14].

Heterogeneity was assessed using the Q-statistic and classified as low, moderate, or high, with thresholds of 25%, 50%, and 75%, respectively. Publication bias was evaluated using a funnel plot when at least 10 studies were included for those outcomes. Subgroup and sensitivity analyses were conducted to assess heterogeneity and confirm the robustness of the results when needed.

### Certainty of evidence

The Grading of Recommendations Assessment, Development, and Evaluation (GRADE) approach was used to evaluate the overall certainty of evidence for each outcome, categorizing it as high, moderate, low, or very low [15,16]. This assessment considered five key factors: risk of bias [17], inconsistency [18], indirectness [19], imprecision [20], and publication bias [21]. The GRADEpro Guideline Development Tool (http://gradepro.org) was used to generate the GRADE evidence profile table and to estimate absolute effects.

## Results

### Search results

The database search identified 3,885 records. After screening titles and abstracts, 41 full-text articles were assessed for eligibility. Twelve studies met the inclusion criteria, and an additional 13 were identified by screening reference lists. In total, 25 RCTs were included in the systematic review and 17 RCTs in the meta-analysis (Fig 1 and S1 Table).

**Fig 1:**
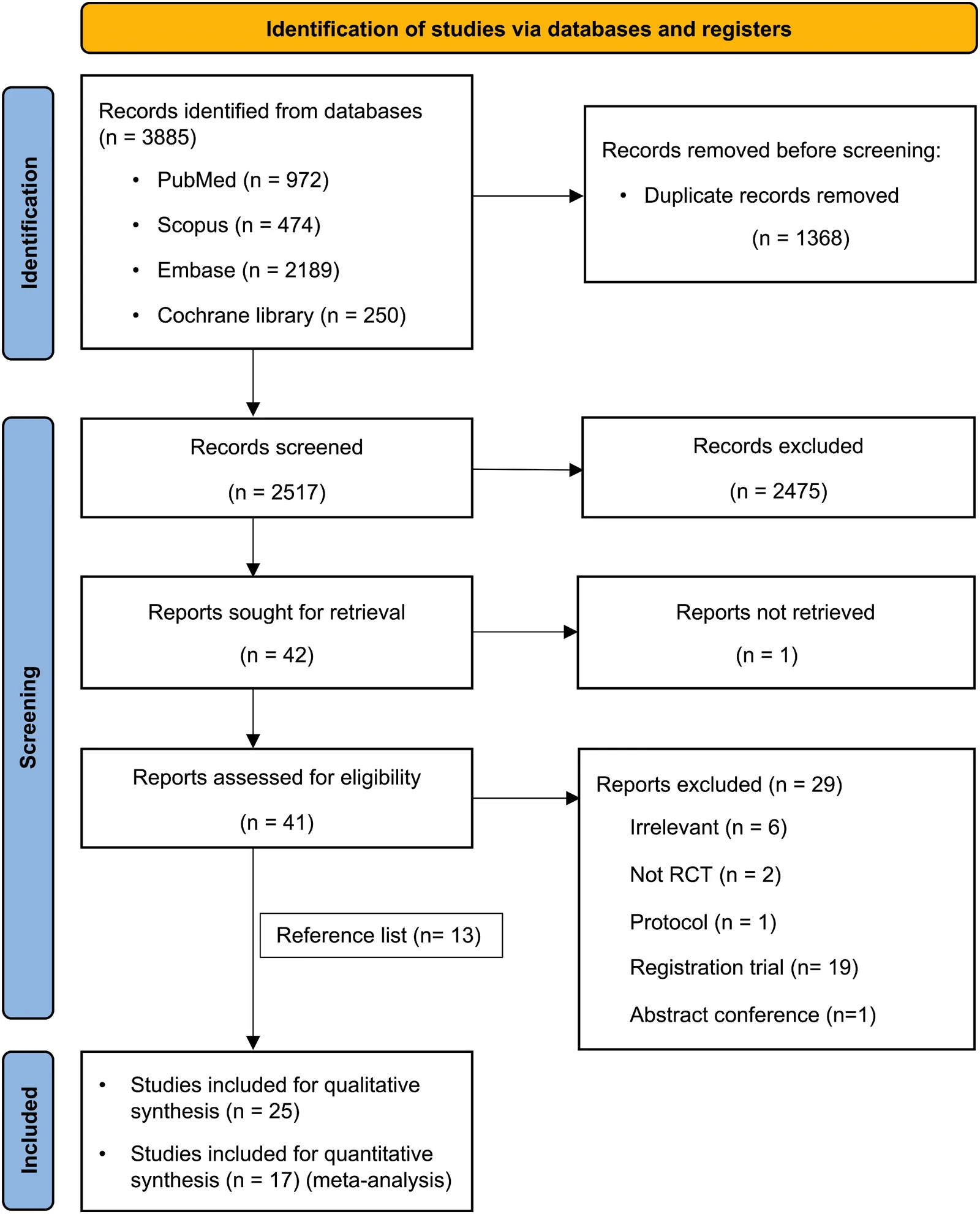
PRISMA flow diagram of the study selection for the systematic review and meta-analysis.

### Study characteristics

The systematic review included 25 RCTs [3,5,9,10, 22–42] while meta-analysis includes 17 studies [5,9,10,22,24–27,29–31,34–39] that involved 1,317 participants with TTN; 663 were treated with nebulized salbutamol, and 654 received placebo and/or standard of care. Single studies were conducted in Turkey [5], Korea [22], and Mexico; [3] two were conducted in India [23,39], seven in Pakistan [32,33,36,38,40–42], seven in Iran [9,10,24–27,34], and six in Egypt [28–31,35,37]. Of the 25 included studies, 22 were conducted in tertiary-care or academic teaching hospitals [5,9,10,22–29,31–37,39–42], two in a secondary-level hospital [3,38], and the setting of one study could not be classified [30].

In 20 studies, salbutamol was administered via nebulization at 0.15 mg/kg/dose [3,5,10,22–30,32,33,37–42]. Other dosing regimens included 0.1 mg/kg/dose [34], 0.9 mg/kg/dose [31], and 2.5 mg/dose [9]. A single study reported 1 mg/dose [36], and another reported 0.15 mg/kg/day [35]. With eight studies giving multiple doses [3,10,25,26,31,32,35,42]. The RCTs included in this review were published between 2011 and 2025. Among the included studies, the clinical score of TTN reported was based on the scoring system used in the original research. Fifteen studies [5,9,26,28–38,42] used the Respiratory Distress Score, 3 studies [23,39,40] used Downes’ Score, and 3 studies [10,24,27] used the Silverman-Anderson Retraction Score. Four studies [3,22,25,41] did not specify a severity score. Of those, five [24,31,34,36,39] reported a mean TTN clinical score <5, while thirteen studies [5,9,10,23,26–30,33,37,38,42] reported a mean TTN clinical score ≥5. The remaining seven studies [3,22,25,32,35,40,41] did not specify TTN clinical scores. Sensitivity analysis was conducted for outcomes from these two studies [9,10], whose mean TTN clinical scores were approximately 5. The study characteristics and participant details are outlined in Table 1 and S2 Table.

**Table 1:** The characteristics of the included studies.

Author, year	Type of study	Location	Inclusion criteria	TTN criteria	Exclusion criteria	Randomization method	Study period	Intervention (n)	Control (n)
**Armangil 2011**	RCT (double blinded)	Turkey	• Diagnosed with TTN • Age < 6 h old	• Onset of tachypnea (>60 breaths/min) within 6 h after birth • Persistence of tachypnea for at least 12 h • CXR findings consistent with TTN • Exclusion of other known respiratory disorders	• Tachypnea due to respiratory (MAS, RDS, pneumonitis) or non-respiratory (hypocalcemia, persistent hypoglycemia, polycythemia) disorder • CHD • Received diuretics and antibiotics	NR	Jan 2007–Jan 2009	Salbutamol at 0.15 mg/kg in 0.9% NSS via NB for one dose (n = 32)	0.9% NSS via NB for one dose (n = 22)
**Kim 2014**	RCT (double blinded)	Korea	• Diagnosed with TTN • Admitted to NICU	• GA ≥ 35 weeks • Symptoms occurred at < 6 h old • CXR findings consistent with TTN	• MAS • Tachypnea due to respiratory (RDS, PPHN, pneumonia) or non-respiratory (EOS, polycythemia, hypoglycemia) disorder • Heart murmur • Tachycardia or arrhythmia	NR	Jan 2010–Dec 2010	Salbutamol at 0.15 mg/kg in 0.9% NSS via NB for one dose (n = 28)	0.9% NSS via NB for one dose (n = 12)
**Monzoy-Ventre 2015**	RCT	Mexico	• GA 34–42 weeks • Diagnosed with TTN • Admitted to NICU	• RR > 60 breaths/min at 6 h old • Symptoms persisted for 12 h • CXR findings consistent with TTN	• Congenital defects • Respiratory disorders (MAS, RDS, pneumonia) • Non-respiratory disorders (EOS)	NR	Feb 2012–Feb 2013	Salbutamol at 0.15 mg/kg in 0.9% NSS via NB every 4 h three times (n = 15)	0.9% NSS via NB every 4 h three times (n = 15)
**Kaur 2017**	RCT (double blinded)	India	• GA 35–41 weeks • Diagnosed with TTN	• Symptoms occurred at < 6 h old • Symptoms persisted • CXR findings consistent with TTN	• GA < 35 weeks • Requiring respiratory support (CPAP, MV) • Tachypnea due to respiratory disorder (MAS, RDS, birth asphyxia history) • CHD	Computer-generated random number table	NR	Salbutamol at 0.15 mg/kg in 0.9% NSS via NB for one dose (n = 50)	0.9% NSS via NB for one dose (n = 50)
**Mohammadzadeh 2017**	RCT (double blinded)	Iran (multicenter)	• GA ≥ 34 weeks • Diagnosed with TTN • Symptoms occurred at < 6 h old	• Respiratory rate > 60 breaths/min with or without cyanosis, respiratory distress • CXR findings consistent with TTN	• GA < 34 weeks • Congenital gross anomalies • MAS • Birth trauma or asphyxia • Chorioamnionitis • History of mother receiving corticosteroids in 7 days before birth • Neonatal sepsis • PPHN • RDS • Metabolic disorder (e.g., hypoglycemia, hypokalemia) • Neonatal cardiovascular disease	NR	Jun 2014–Dec 2014	Salbutamol at 0.15 mg/kg in 0.9% NSS via NB (n = 35)	0.9% NSS via NB (n = 35)
**Mussavi 2017**	RCT (blinded)	Iran	• Diagnosed with TTN • Respiratory distress score > 4 to < 10	• GA ≥ 35 weeks • Symptoms occurred at < 6 h old • Typical TTN chest radiography findings	• MAS • Tachypnea due to respiratory (RDS, PPHN, pneumonia) or non-respiratory (sepsis, hypoglycemia, polycythemia) disorder • Birth asphyxia history • CHD • Tachycardia or arrhythmia • Respiratory distress score < 5 or > 10	Computer generated	Aug 2015–Dec 2015	Salbutamol at 0.15 mg/kg in NSS via NB every 6 h for 24 h (n = 30)	NSS via NB every 6 h for 24 h (n = 30)
**Malakian 2018**	RCT (triple blinded)	Iran	• Diagnosed with TTN	• Respiratory rate > 60 breaths/min • Symptoms occurred at < 6 h old • CXR findings consistent with TTN	• Requiring respiratory support (MV) • Congenital malformation • Perinatal asphyxia • Hypocalcemia • Systemic infection • MAS • RDS • IUGR • History of fetal distress • Pneumonitis • CHD • DIC • Multiorgan failure • Hypoxemia • Hypoglycemia • Polycythemia	Table of random numbers	NR	Salbutamol at 0.15 mg/kg via NB every 6 h for a maximum of 72 h if respiratory distress persisted (n = 74)	NSS via NB every 6 h for a maximum of 72 h if respiratory distress persisted (n = 74)
**Babaei 2019**	RCT	Iran	• GA ≥ 35 weeks • Diagnosed with TTN • Admitted to NICU	• Symptoms occurred at < 6 h old • CXR findings consistent with TTN	• MAS • RDS • Congenital pneumonia • Polycythemia • Hypoglycemia • EOS • Cardiac disorder • Tachycardia • Arrhythmia • Congenital anomaly	Random number table	During 2017	Salbutamol at 0.15 mL/kg (0.15 mg/kg/dose) in NSS via NB for one dose (n = 40)	0.9% NSS via NB for one dose (n = 40)
**Al Lahony 2020**	RCT	Egypt	• GA > 36 weeks • Postnatal age < 6 h at time of enrollment • TTN	• CXR findings consistent with TTN	NR	NR	Mar 2017–Dec 2017	Salbutamol at 0.15 mg/kg in NSS via NB (n = 30)	0.9% NSS via NB for one dose (n = 30)
**Salama 2020**	RCT	Egypt	• GA 35–39 weeks • Delivered via C/S or vaginal delivery • Diagnosed with TTN	• Physical examination findings suggest TTN • CXR findings consistent with TTN	• RDS • Pneumonia • MAS • CHD • Hypoglycemia • Polycythemia • EOS • Tachycardia • Arrhythmia • Congenital anomaly • Polycythemia	NR	Jul 2018–Apr 2019	Salbutamol at 0.15 mg/kg in NSS via NB for one dose (n = 50)	0.9% NSS via NB for one dose (n = 50)
**Talaat 2020**	RCT (blinded)	Egypt (multicenter)	• Admitted to intermediate care or NICU • Diagnosed with TTN • Age < 6 h old	• Symptoms occurred at < 6 h old • Symptoms persisted for at least 12 h • CXR findings consistent with TTN	• Tachypnea due to respiratory (RDS, MAS, pneumonitis) or non-respiratory (CHD, hypoglycemia, polycythemia) disorder	NR	2018–2019	Salbutamol at 0.15 mg/kg in NSS for one dose via NB (n = 54)	0.9% NSS for one dose via NB (n = 46)
**El-Badawy 2021**	RCT	Egypt	• Full term • TTN	• Symptoms occurred within 24 h of birth • Symptoms persisted for 12–24 h or < 72 h • Spontaneous improvement • CXR findings consistent with TTN	• Symptoms occurred > 24 h after birth • Severe respiratory distress with cyanosis • CHD • Sepsis • Inborn error of metabolism • Congenital lung malformation • Chromosomal disorder	NR	Dec 2018–Jun 2020	Salbutamol at 0.15 mL/kg (0.9 mg/kg/dose) in NSS via NB every 8 h until clinical resolution (n = 25)	0.9% NSS via NB every 8 h until clinical resolution (n = 25)
**Maroof 2021**	RCT	Pakistan	• GA > 37 weeks • Diagnosed with TTN	• Respiratory rate > 60 breaths/min • Symptoms occurred at < 6 h old • Silverman–Anderson score > 5	• RDS• MAS • CHD • Congenital pneumonia • PPHN • EOS/DIC• Hypoglycemia	Lottery	Aug 2017–Feb 2018	Salbutamol at 0.15 mg/kg in NSS via NB every 4 h for two doses (n = 50)	0.9% NSS via NB every 4 h for two doses (n = 50)
**Ahmed 2022**	RCT (double blinded)	Pakistan	• GA ≥ 37 weeks • Delivered via C/S • TTN score ≥ 4 • Respiratory rate > 60 breaths/min • Symptoms occurred at < 6 h old	• CXR findings consistent with TTN	• Maternal history of premature rupture of membranes > 18 h • Maternal history of PIH, GDM • MASF • No ANC • Twin pregnancy	NR	Jan 2017–Mar 2018	Salbutamol at 0.15 mg/kg in NSS via NB (n = 30)	0.9% NSS via NB (n = 30)
**Basiri 2022**	RCT (double blinded)	Iran	• GA 34–37 weeks • Respiratory rate > 60 breaths/min • Symptoms occurred at < 6 h old and persisted for 12 h • Diagnosed with TTN	• CXR findings consistent with TTN	• RDS • MAS • Sepsis • Focal infiltration on CXR • Tachypnea due to non-respiratory disorder (hypocalcemia, persistent hypoglycemia, polycythemia) • CHD • History of maternal substance abuse • Death during the study	Permuted block randomization method	NR	Salbutamol at 0.1 mg/kg in 0.9% NaCl via NB (n = 26)	0.9% NaCl via NB (n = 26)
**Hamed 2022**	RCT	Egypt	• GA > 36 weeks • Diagnosed with TTN	• Clinically consistent with TTN • CXR findings consistent with TTN	• CHD • MAS • Sepsis • Pneumonia	NR	NR	Salbutamol at 0.15 mg/kg/day via NB every 6 h for 72 h (n = 50)	None (n = 50)
**Khushdil 2022**	RCT	Pakistan	• Neonates • Diagnosed with TTN	• Clinically consistent with TTN • CXR findings consistent with TTN	• Vaginal delivery • Major congenital malformation • History of meconium aspiration • EOS • CHD	Computer generated	Oct 2019–May 2020	Salbutamol (5 mg/mL) at 0.2 mL in 0.9% NSS via NB (n = 25)	None (n = 25)
**Sabry 2022**	RCT (blinded)	Egypt	• GA 35–39 weeks • Admitted to NICU due to persistent tachypnea at 2–6 h of life that persisted for at least 12 h • Diagnosed with TTN • Delivered via C/S or vaginal delivery	• Symptoms occurred at 2–6 h old and persisted for at least 12 h • CXR findings consistent with TTN	• RDS • Sepsis • Pneumonia • Severe pulmonary hypertension • MAS • CHD • Perinatal asphyxia • Congenital malformation • Non-respiratory disorder • Death in the first 24 h after enrollment	Random number table sequence	1 year period	Salbutamol at 0.15 mg/kg via NB for one dose (n = 25)	None (n = 50)
**Anwar 2023**	RCT (open label)	Pakistan	• GA ≥ 34 weeks • Admitted to NICU • Diagnosed with TTN	• GA ≥ 34 weeks • Symptoms occurred within 6 h of life • CXR findings consistent with TTN	• APGAR score at 5 min ≤ 6 • MAS • Hypocalcemia • Hypoglycemia • Polycythemia • CHD • Tachycardia during NB • Major congenital malformation • IUGR • RDS • Emergency C/S • Requiring ventilation and CPAP support • Sepsis	NR	Jan 2018–Nov 2022	Salbutamol at 0.15 mg/kg via NB for one dose (n = 40)	None (n = 40)
**Choobdar 2024**	RCT (triple blinded)	Iran	• GA > 35 weeks • Birth weight > 2 kg • Diagnosed with TTN	• Symptoms occurred within 6 h of life • CXR findings consistent with TTN	• MAS • RDS • PPHN • EOS • Focal infection on CXR • Polycythemia • Hyperglycemia • Hypocalcemia • Heart murmur • Tachycardia • Arrhythmia • Asphyxia	Blocked randomization method	Nov 2022–Jan 2023	Salbutamol 2.5 mg via NB for one dose (n = 30)	NSS for one dose via NB (n = 30)
**Dhaka** **2024**	RCT (double blinded)	India	• GA ≥ 34 weeks • Diagnosed with TTN	• Symptoms occurred within 6 h of life • Clinically consistent with TTN • CXR findings consistent with TTN	• Tachypnea due to respiratory (RDS, MAS, pneumonia) or non-respiratory (hypocalcemia, persistent hypoglycemia, polycythemia) disorder • CHD • Neonates with maternal risk factors for sepsis/chorioamnionitis, perinatal asphyxia • Major congenital and/or chromosomal anomalies	Computer-based variable block random sequence	May 2023–Jul 2024	Salbutamol at 0.15 mg/kg in 0.9% NSS via NB for one dose (n = 67)	0.9% NSS via NB for one dose (n = 67)
**Alvi 2025**	RCT	Pakistan	• GA > 35 weeks • Diagnosed with TTN	• Symptoms occurred within 6 h of life • CXR findings consistent with TTN	• History of meconium aspiration • Congenital pneumonia • Polycythemia • Hypoglycemia • Cardiac disorders • Tachycardia or arrhythmia	Non-probability consecutive sampling	Jun 2023–Dec 2023	Salbutamol at 0.15 mg/kg in 0.9% NSS via NB for one dose (n = 30)	0.9% NSS via NB for one dose (n = 30)
**Gooran 2025**	RCT	Iran	• GA > 34 weeks • Diagnosed with TTN	• Clinically consistent with TTN • CXR findings consistent with TTN	• MAS • Complex CHD • Tachycardia (heart rate >180 beats per minute) • RSS < 4 • Need for respiratory sup- port with a ventilator after birth • Sepsis • RDS	• Permuted block randomization method with blocks of 4	Mar 2022–Apr 2023	Salbutamol at 0.15 mg/kg in NSS via NB q 6 h for 1 day (n = 32)	0.9% NSS via NB q 6 h for 1 day (n = 32)
**Hussain 2025**	RCT	Pakistan	• Neonates of either gender with a respiratory rate > 60 breaths/min, without any obvious cause, and not settled within the first 60 minutes of life	NR	• GA < 35 weeks • CHD • MSAF • Opacities in lung fields seen in CXR • Clinical manifestations of EOS • Maternal fever or prolonged rupture of membrane (>18 h) • Birth asphyxia • Hypoglycemia within 1 h of life • Adverse effects: tachycardia or hypoglycemia that might limit the trial’s progression	Random and unbiased selection of participants	Sep 2024–Nov 2024	Salbutamol at 0.15 mg/kg in NSS via NB, if tachypnea persisted, a second dose was administered (n = 30)	NSS via NB, if tachypnea persisted, a second dose was administered (n = 30)
**Ullah 2025**	RCT	Pakistan	• GA > 36 weeks • Diagnosed with TTN	• Symptoms occurred within 6 h of life • TTN score ≥ 4 using the modified Downes score • CXR findings consistent with TTN	• MSAF • PIH • GDM gestational diabetes mellitus (fasting • Pre-existing renal disease or liver disease • Maternal history of premature rupture of membranes >18 hours • Unbooked antenatal status	Blocked randomization method	Aug 2024 –Dec 2024	Salbutamol at 0.15 mg/kg in NSS via NB for one dose (n = 30)	0.9% NSS via NB for one dose (n = 30)

ANC, antenatal care; BW, birth weight; CDH, congenital diaphragmatic hernia; CHD, congenital heart disease; CPAP, continuous positive airway pressure; C/S, cesarean section; CXR, chest X-ray; DIC, disseminated intravascular coagulation; EOS, early onset sepsis; GA, gestational age; GDM gestational diabetes, IUGR intrauterine growth retardation, MAS meconium aspiration syndrome, MASF meconium stained amniotic fluid, MV, mechanical ventilation; NB, nebulization; NICU, neonatal intensive care unit; NR, no report; NSS, normal saline solution PIH, pregnancy-induced hypertension; PPHN, persistent pulmonary hypertension of the newborn; RCT, randomized controlled trial; RDS, respiratory distress syndrome; RR, respiratory rate; RSS, Silverman-Andersen respiratory severity score; TTN, transient tachypnea of the newborn.

### Risk of bias assessment

Of the 25 included studies, only 5 used appropriate methods for generating allocation sequences and implementing allocation concealment [9,10,34,37,39]. All 25 studies implemented the intended interventions and adequately accounted for loss to follow-up. Outcome measurements were deemed appropriate in 4 studies [5,9,26,39], and results reporting was adequate in all studies. According to the revised Cochrane risk-of-bias assessment, 2 studies were classified as having low risk of bias [9,39], while the remaining 23 were categorized as having some concerns (S1 Fig).

### Data analyses

Figs 2–4, Table 2, S2 Fig, and S3 Table demonstrate that salbutamol improves clinical outcomes in infants with TTN for certain respiratory parameters, time to initiate feeding, and duration of hospitalization. However, in subgroup analyses stratified by TTN severity, classified as a mean TTN clinical score of <5 or ≥5 as defined in the original studies, no significant differences were found between infants receiving salbutamol and those receiving standard care or placebo in the subgroup with a mean TTN clinical score <5. Table 2 summarizes the certainty of the evidence according to the GRADE approach.

**Table 2:** The Grading of Recommendations Assessment, Development, and Evaluation (GRADE) summary of the findings on the efficacy of salbutamol in infants with transient tachypnea of the newborn.

Patient or population: neonates Intervention: salbutamol Control: placebo and/or standard of care										
No. of studies	Certainty assessment					No. of participants		Effect		
	Risk of bias	Inconsistency	Indirectness	Imprecision	Other considerations	Salbutamol	Placebo and/or standard of care	Estimation of a solute effects		Certainty
								Relative (95% CI)	Absolute (95% CI)	
**Respiratory outcomes**										
**Duration of tachypnea (hours)**										
3	serious^a^	serious^b^	not serious	serious^c^	none	135	119	–	MD 9.93 **lower**(25.42 lower to 5.56 higher)	⨁◯◯◯ Very low
**Duration of respiratory support (hours)**										
6	serious^a^	serious^b^	not serious	not serious	none	236	218	–	MD **14.85 lower**(25.05 lower to 4.65 lower)	⨁⨁◯◯ Low
**Duration of respiratory support (hours) in TTN clinical score <5**										
3	serious^a^	serious^b^	not serious	serious^c^	none	118	118	–	MD **10.88 lower**(24.48 lower to 2.73 higher)	⨁◯◯◯ Very low
**Duration of respiratory support (hours) in TTN clinical score ≥5**										
3	serious^a^	serious^b^	not serious	not serious	none	118	100	–	MD **20.19 lower**(34.88 lower to 5.49 lower)	⨁⨁◯◯ Low
**Duration of continuous positive airway pressure (hours)**										
4	serious^a^	serious^b^	not serious	not serious	none	169	194	–	MD **18.19 lower**(30.03 lower to 6.35 lower)	⨁⨁◯◯ Low
**Duration of oxygen requirement (hours)**										
8	serious^a^	serious^b^	not serious	not serious	none	310	306	–	MD **24.57 lower**(35.03 lower to 14.1 lower)	⨁⨁◯◯ Low
**Duration of oxygen requirement (hours) in TTN clinical score <5**										
2	serious^a^	not serious	not serious	serious^c^	none	60	60	–	MD **9.02 lower**(19.48 lower to 1.45 higher)	⨁⨁◯◯ Low
**Duration of oxygen requirement (hours) in TTN clinical score ≥5**										
4	serious^a^	serious^b^	not serious	not serious	none	179	204	–	MD **20.75 lower**(25.72 lower to 15.79 lower)	⨁⨁◯◯ Low
**Time to initiate feeding (hours)**										
11	serious^a^	serious^b^	not serious	not serious	publication bias strongly suspected^d^	457	466	–	MD **7.16 lower**(11.77 lower to 2.55 lower)	⨁◯◯◯ Very low
**Duration of hospitalization (days)**										
13	serious^a^	serious^b^	not serious	not serious	publication bias strongly suspected^d^	516	482	–	MD **1.17 lower**(1.69 lower to 0.65 lower)	⨁◯◯◯ Very low
**Duration of hospitalization (days) in TTN clinical score <5**										
3	serious^a^	serious^b^	not serious	serious^c^	none	86	86	–	MD **0.2 lower**(0.96 lower to 0.55 higher)	⨁◯◯◯ Very low
**Duration of hospitalization (days) in TTN clinical score ≥5**										
8	serious^a^	serious^b^	not serious	not serious	none	352	334	–	MD **1.47 lower**(2.17 lower to 0.78 lower)	⨁⨁◯◯◯ Low
**Duration of neonatal intensive care unit stay (days)**										
2	serious^a^	serious^b^	not serious	serious^c^	none	92	117	–	MD **0.84 lower**(2.62 lower to 0.94 higher)	⨁◯◯◯ Very low

^a^Downgraded by one level due to risk of bias concerns related to the randomization process and outcome assessment.^b^ Downgraded by one level due to inconsistency, reflected in substantial heterogeneity (I² = 50%).^c^ Downgraded by one level for imprecision because the 95% confidence interval suggests both potential benefit and harm.

^d^Downgraded by one level for publication bias due to asymmetry observed in the funnel plot, suggesting potential selective reporting of positive results.

CI, confidence interval; MD, mean difference; RCTs, randomized controlled trials; TTN, transient tachypnea of the newborn.

**Fig 2:**
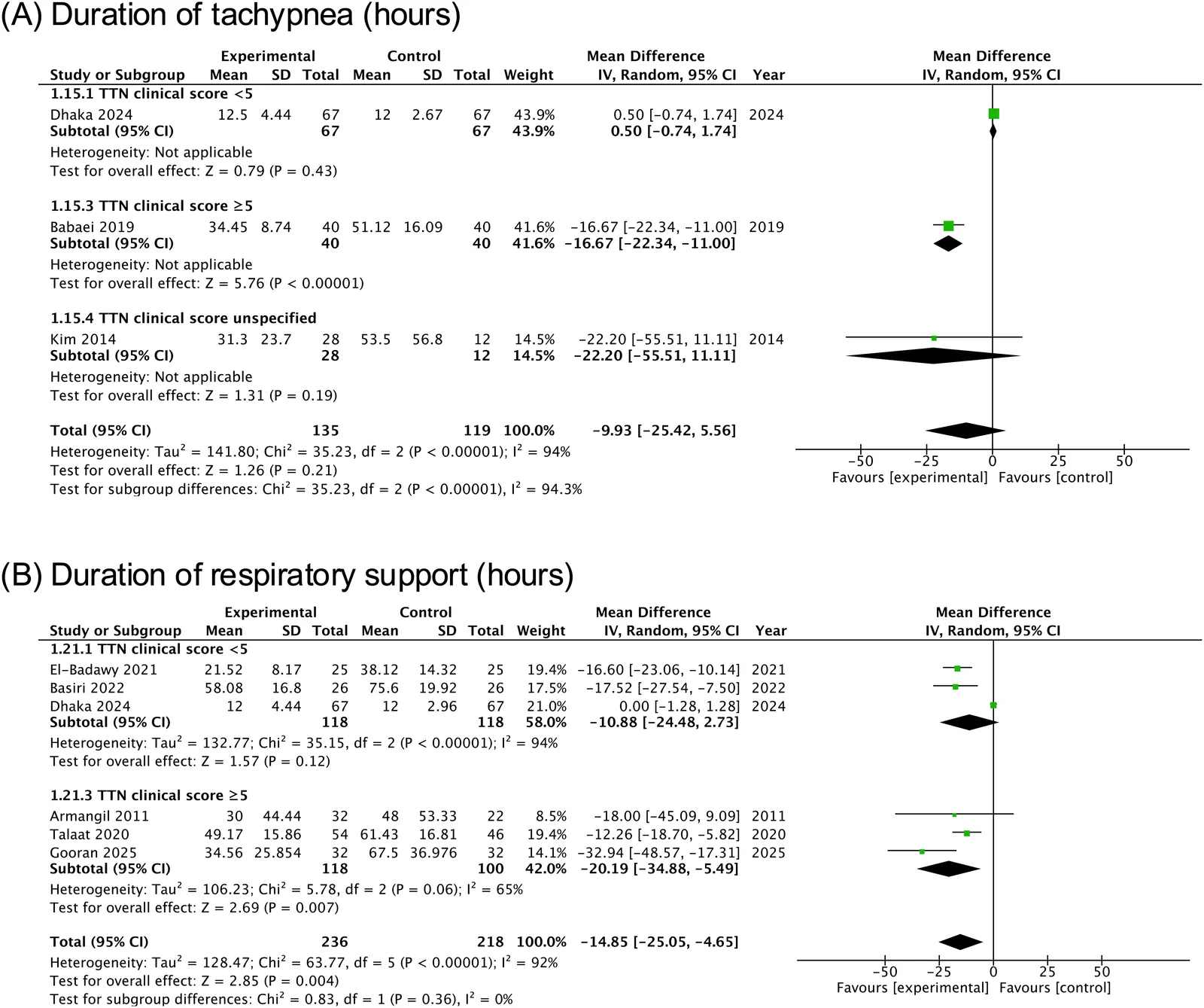
Forest plots of the efficacy of salbutamol in infants with TTN. (A) Duration of tachypnea (hours), (B) Duration of respiratory support (hours).

**Fig 3:**
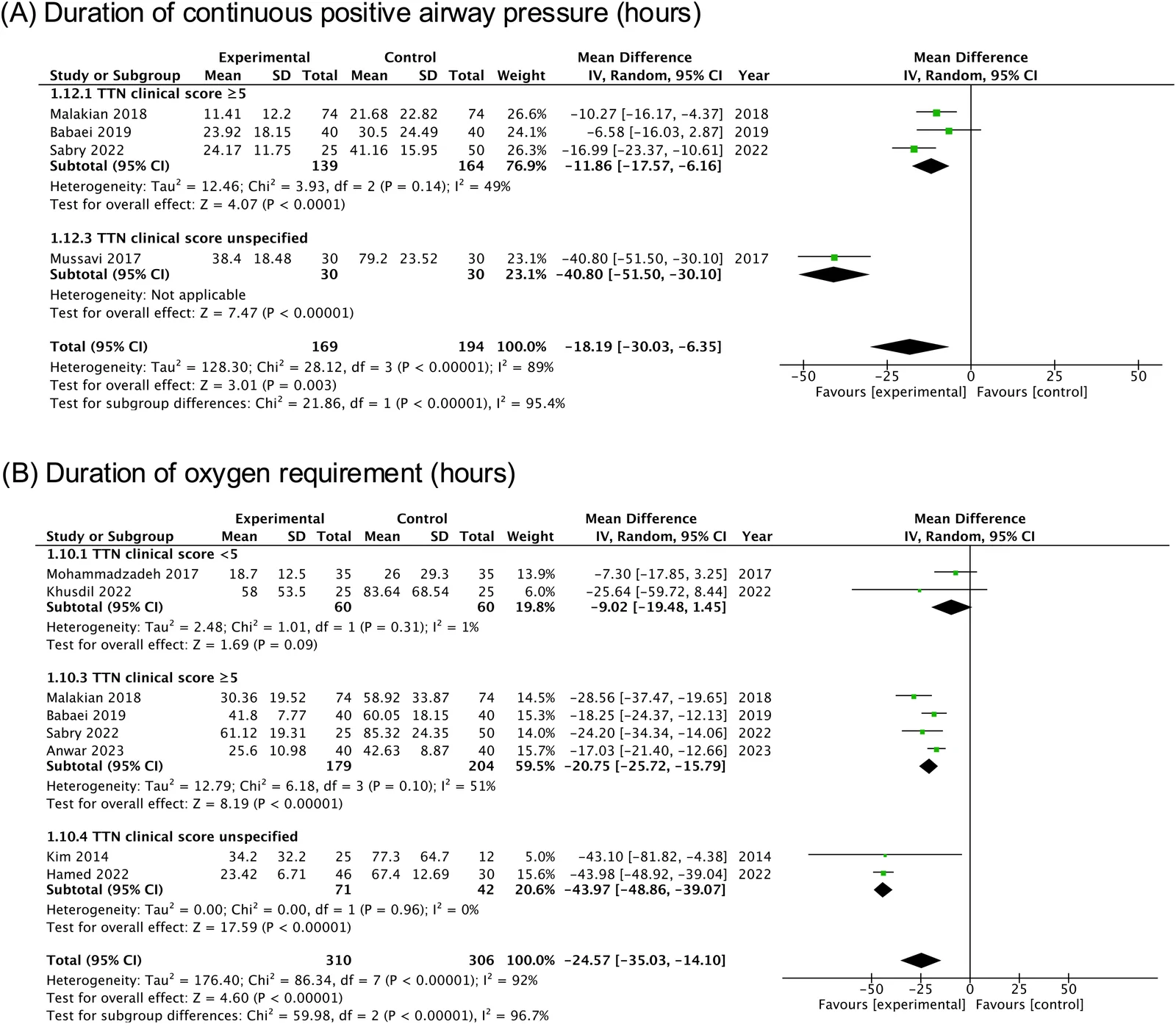
Forest plots of the efficacy of salbutamol in infants with TTN. (A) Duration of continuous positive airway pressure (hours), (B) Duration of oxygen requirement (hours).

**Fig 4:**
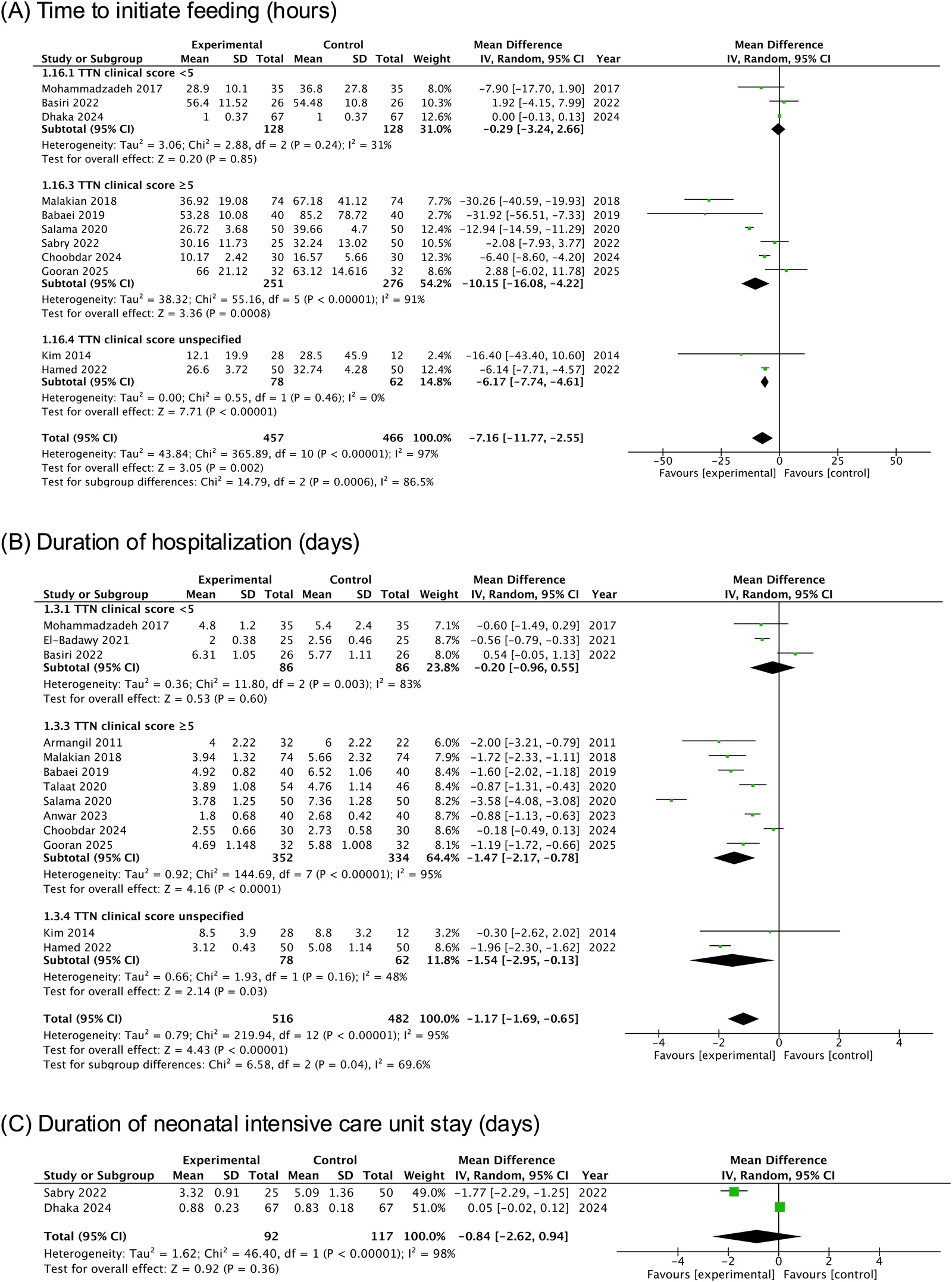
Forest plots of the efficacy of salbutamol in infants with TTN. (A) Time to initiate feeding (hours), (B) Duration of hospitalization (days), (C) Duration of neonatal intensive care unit (NICU) stay (days).

### Respiratory outcomes

#### Duration of tachypnea (hours).

Three studies [22,27,39] reported the duration of tachypnea in a total of 254 infants diagnosed with TTN. The duration of tachypnea did not differ significantly between the treatment and control groups (MD −9.93 hours; 95% CI −25.42 to 5.56; p = 0.21). For this outcome, statistical heterogeneity was high (I² = 94%) and the certainty of the evidence was rated as very low (Fig 2A, Table 2, S2A Fig, and S3 Table).

### Duration of respiratory support (hours)

When we analyzed data from six RCTs [5,10,30,31,34,39], which involved 454 participants, the results revealed that the duration of respiratory support was significantly shorter in the treatment group compared to the control group (MD: −14.85 h; 95% CI [−25.05, −4.65]; p = 0.004). High heterogeneity in this outcome was observed (I^2^ = 92%). The certainty of the evidence for this outcome was rated as low. In the subgroup analysis classified by TTN clinical score, there was no significant difference between the salbutamol group and the control group among studies with a TTN clinical score of <5 (MD: −10.88 h; 95% CI [−24.48, 2.73]; p = 0.12) (Fig 2B, Table 2, S2B Fig, and S3 Table). Sensitivity analysis excluding Gooran et al. [10] from the pooled meta-analysis in the TTN clinical score ≥5 subgroup did not alter the statistical significance of this outcome (MD: −11.73 h; 95% CI [−21.74, −1.73]; p = 0.02) (S4A Fig).

### Duration of continuous positive airway pressure (CPAP) (hours)

In an analysis of data from four RCTs [25–27,37] involving 363 participants, the duration of CPAP was found to be notably shorter in the salbutamol group compared to the control group (MD: −18.19 h; 95% CI [−30.03, −6.35]; p = 0.003). Substantial heterogeneity was observed (I² = 89%). The certainty of the evidence was rated as low (Fig 3A, Table 2, S2C Fig, and S3 Table).

### Duration of oxygen requirement (hours)

In terms of the time of receiving oxygen therapy, the analysis of data from 8 RCTs [22,24,26,27,35–38], involving 616 participants, showed that the duration of oxygen supplementation in the salbutamol group was significantly shorter than that in the control group (MD: −24.57 h; 95% CI [−35.03, −14.10]; p < 0.001). High heterogeneity in this outcome was noticed (I^2^ = 92%). However, subgroup analysis revealed no significant difference in studies with a mean TTN clinical score of <5 group (MD: −9.02 h; 95% CI [−19.48, 1.45]; p = 0.09). The certainty of the evidence was rated as low (Fig 3B, Table 2, S2D Fig, and S3 Table).

### Time to initiate feeding (hours)

Analysis of data from 11 RCTs [9,10,22,24,26,27,29,34,35,37,39], which involved 923, to assess the time to initiation of feeding revealed that feeding was started considerably earlier in the salbutamol group compared to the control group (MD: −7.16 h; 95% CI [−11.77, −2.55]; p = 0.002). The funnel plot indicated the presence of publication bias for this outcome (S3A Fig). High heterogeneity was observed (I^2^ = 97%). The certainty of the evidence was rated as very low (Fig 4A, Table 2, S2E Fig, and S3 Table). Sensitivity analyses excluding Choobdar et al. [9] and Gooran et al. [10] from the pooled meta-analysis in the TTN clinical score ≥5 subgroup did not alter the statistical significance of this outcome (MD: −8.55 h; 95% CI [−14.04, −3.06]; p = 0.002) (S4B Fig).

### Duration of hospitalization stay (days)

We analyzed data from 13 RCTs [5,9,10,22,24,26,27,29–31,34,35,38], which involved 998 participants, to assess the duration of hospitalization. The salbutamol group had a notably shorter length of hospital stay compared to the control group (MD: −1.17 days; 95% CI [−1.69, −0.65]; p < 0.001). The funnel plot indicated the presence of publication bias for this outcome. High heterogeneity was observed in this outcome (I^2^ = 95%). Subgroup analysis of studies with a TTN clinical score of <5 showed no significant difference between the salbutamol group and the control group (MD: −0.20 days; 95% CI [−0.96, −0.55]; p = 0.60). The certainty of the evidence was rated as very low. The funnel plot indicated the presence of publication bias for this outcome. (Fig 4B, Table 2, and S2F Fig, S3 Table, and S3B Fig).

Sensitivity analyses excluding Choobdar et al. [9] and Gooran et al. [10] from the pooled meta-analysis in the TTN clinical score ≥5 subgroup did not alter the statistical significance of this outcome (MD: −1.27 days; 95% CI [−1.86, −0.68]; p < 0.001) (S4C Fig).

### Duration of neonatal intensive care unit (NICU) stay (days)

We analyzed data from 2 RCTs [37,39], which involved 209 participants, to assess the duration of NICU stay. The results showed that the salbutamol group had not significantly decreased in the length of stay compared to the control group (MD: −0.84 days; 95% CI [−2.62, 0.94]; p = 0.36). Considerable heterogeneity in this outcome was noticed (I^2^ = 98%). The certainty of the evidence was rated as very low (Fig 4C, Table 2, S2G Fig, and S3 Table).

## Discussion

Our systematic review and meta-analysis presents updated evidence on the use of salbutamol for the management of TTN. A total of 25 RCTs were included in the systematic review, of which 17 were included in the quantitative meta-analysis. Eight studies were not included in the pooled estimate because their outcomes, such as respiratory rate, SpO_2_, or TTN clinical score, were reported only as pre- and post-intervention measurements, making direct comparison across studies infeasible, as baseline severity differed across studies. In addition, some of these studies reported results in formats incompatible with pooled analysis, so they could not be analyzed in the pooled analysis.

Among the 17 RCTs included in the meta-analysis, salbutamol was associated with significant improvements in respiratory outcomes, including duration of respiratory support, duration of CPAP use, duration of oxygen supplementation, time to initiation of feeding, and length of hospital stay. However, two outcomes, duration of tachypnea and duration of NICU stay, did not show significant improvement in the salbutamol group compared to the control group.

Although these outcomes were statistically significant, high heterogeneity was observed across all outcomes; therefore, subgroup analysis was performed. The severity of TTN was used to stratify studies based on clinical TTN scoring systems. As mentioned earlier, three different scoring systems were used across the included studies: the Respiratory Distress Score (used in 15 of 25 studies), the Downes’ Score (3 studies), and the Silverman–Anderson Retraction Score (3 studies). The remaining four studies did not report a TTN severity score and were therefore classified as the unidentified TTN scoring group. Studies were subgrouped according to mean TTN clinical score into two categories: < 5 and ≥5.

Following subgroup analysis, heterogeneity decreased in the outcomes of CPAP duration, duration of oxygen requirement, and time to initiate feeding. Notably, studies with a mean TTN clinical score of <5 showed no significant difference between the salbutamol group and the control group in the outcomes of respiratory support duration, oxygen supplementation duration, time to initiation of feeding, and length of hospital stay. Notably, pooled meta-analyses of studies with a mean TTN clinical score ≥5 showed a significant benefit of salbutamol over the control group across those outcomes, including CPAP duration, as well as for some outcomes in studies with an unspecified TTN clinical score.

While the outcome of duration of tachypnea was not significant in our meta-analysis, the pooled analysis for this outcome included three studies, which differed in severity. Therefore, the result for this outcome may have some degree of limitation in the comparison of tachypnea duration.

Besides that, the recovery time exceeded the typical 48–72-hour window within which TTN usually resolves, raising concern that the diagnosis may have reflected another condition rather than TTN. In addition, the timing of excluding early-onset sepsis raises a separate concern. However, upon reviewing the inclusion criteria of the included studies, we found them to be consistent with TTN and to have excluded other causes as possible, as specified in the studies. The longer resolution time observed in some studies may instead reflect greater disease severity, with recovery taking longer than typical and requiring a higher level of respiratory support [43].

A previous meta-analysis by Aznaran-Torres et al. (2025) [44] demonstrated the benefits of salbutamol compared to the control group in neonates with TTN, particularly in respiratory outcomes and length of hospital stay. Our findings were also consistent with this previous meta-analysis. However, two outcomes, duration of tachypnea and duration of NICU stay, did not show significant improvement with salbutamol. This discrepancy may be partly explained by the fact that we analyzed NICU stay duration separately from overall hospital length of stay. Another previous meta-analysis by Moresco et al. [45] also reported similar benefits of salbutamol in reducing oxygen therapy duration, need for respiratory support, and hospital length of stay. Nonetheless, they cautioned that the low-quality evidence limits definitive conclusions regarding salbutamol’s efficacy and safety in TTN, a limitation that aligns with our own findings.

Furthermore, our subgroup analysis revealed an additional finding not reported in the previous meta-analysis. Salbutamol did not significantly improve outcomes in infants with a mean TTN clinical score <5. This suggests that although salbutamol appears beneficial in the overall pooled analysis, its benefit may be limited to infants with more severe TTN. Accordingly, salbutamol use may be better reserved for higher-severity cases rather than applied routinely across all infants with TTN.

Given the high heterogeneity in the pooled meta-analysis, the effect of salbutamol was not consistently beneficial across all studies. Our findings indicate that disease severity is one such factor. TTN results from inadequate neonatal lung-fluid clearance and retained pulmonary fluid. Although ENaC-mediated sodium absorption is a major mechanism of lung-fluid clearance, its function is influenced by factors such as gestational age and the developmental regulation of ENaC expression [4,46]. Animal studies show that inactivation of the α-subunit of ENaC causes defective lung-fluid clearance, providing direct evidence that ENaC-mediated sodium absorption is a critical, rate-limiting step in neonatal lung-fluid removal [47]. However, the relationship between ENaC genetics and neonatal lung disease is complex. Lung-fluid clearance is also influenced by lung expansion after birth, pulmonary blood flow and lymphatic drainage, mechanical forces during vaginal delivery, and adequate surfactant function [48]. Therefore, it may be challenging to predict the functional status of ENaC in lung-fluid clearance in infants with TTN.

Another hypothesis is that, in mild cases, this delay in ENaC function is minimal, and as TTN follows its natural course, endogenous ENaC-mediated fluid clearance improves over time [48], such that the benefit of pharmacologic intervention may not be readily apparent. In more severe TTN, however, stimulation of β-adrenergic receptors by β2-adrenergic agonists upregulates alveolar epithelial Na⁺ transport by increasing the activity of ENaC and Na ⁺ /K ⁺ -ATPase and increasing their protein abundance at the plasma membrane [6,49]. This pharmacologically driven augmentation may meaningfully compensate for delayed or attenuated channel function in more severe TTN, though non-ENaC mechanisms, such as elevated pulmonary vascular resistance or surfactant deficiency, may also contribute to greater severity. Consequently, β2-agonist therapy may only improve the ENaC-related component of the disease process. This hypothesis, however, remains speculative unless future studies directly measure ENaC function in relation to disease severity.

While the dosage varied across the included studies, and fewer studies used multiple-dose regimens than single-dose regimens, it remains unclear whether single-dose or multiple-dose forms meaningfully influence treatment outcomes.

Other studies included in the systematic review, Al Lahony et al. [28], Ahmed et al. [33], Alvi et al. [41], and Hussain et al.[42], all in the TTN clinical score ≥5 subgroup, reported improved respiratory rate with salbutamol at 4 hours, while Maroof et al. [32] and Monzoy-Ventre et al. [3] reported the same result without a specified severity classification. In contrast, Kaur et al. [23], also in the TTN clinical score ≥5 subgroup, found no significant improvement in respiratory rate or oxygen saturation at 4 hours after treatment. Ullah et al. [40] and Al Lahony et al. [28] reported a reduction in TTN clinical scores with salbutamol use. Regarding safety, Kaur et al. [23] found no significant increase in tachycardia. Al Lahony et al. [28] similarly reported no significant increase in tachycardia, as well as no significant decrease in serum potassium, addressing common safety concerns associated with salbutamol use.

### Strengths and limitations

This study uniquely examined the impact of salbutamol on various clinical outcomes in infants with TTN, including duration of respiratory support, length of hospitalization or NICU stay, and time to initiation of feeding. However, several limitations should be acknowledged. First, small sample sizes and protocol variations across institutions, such as differences in intervention timing and supportive care practices, may have influenced the results. Second, the overall certainty of the evidence was rated as low to very low for most outcomes, and evidence for key outcomes such as time to initiation of feeding and length of hospital stay was further downgraded due to possible publication bias. Third, although our systematic review included a larger number of studies than previous evaluations of salbutamol’s effects in TTN management, important methodological limitations remain. Some included trials showed concerns related to bias in randomization and outcome measurement, leading to an overall risk-of-bias judgment of “some concerns” for most studies. Fourth, because severity was defined using each study’s mean TTN clinical score, classification for some infants with scores near the borderline of 5 may not be entirely precise. We acknowledged this issue and performed a sensitivity analysis to address it. Fifth, the included studies were conducted in only limited geographic regions, which may not represent TTN cases in other parts of the world. Sixth, standardizing a universal treatment strategy is highly challenging due to the clinical variety and heterogeneity of TTN. In fact, as shown clearly in the tables, baseline TTN characteristics vary considerably across the included trials. These limitations highlight the need for further well-designed RCT studies to strengthen the evidence base and to better inform the clinical application of salbutamol. Therefore, future trials would benefit from several methodological improvements. Salbutamol dosing protocols should be standardized, including whether a single-dose or multiple-dose regimen is used. Diagnostic criteria should ensure comparable disease severity across enrolled populations. Larger, multicenter designs are needed to address the limitations of the predominantly small, single-center studies conducted to date. Adverse events should also be recorded systematically, using consistent definitions and timing across trials. Finally, future studies should be conducted across a broader range of geographic settings, rather than remaining concentrated in a limited number of regions, to strengthen the generalizability and reliability of conclusions regarding salbutamol’s benefit in TTN.

## Conclusions

Nebulized salbutamol may improve respiratory outcomes in infants with TTN, including shorter durations of respiratory support, CPAP use, oxygen supplementation, and hospital stay, as well as earlier feeding initiation, though the efficacy of the intervention may be restricted to some severity groups of TTN infants, particularly the more severe cases. However, this evidence remains of low to very low certainty, and further randomized controlled trials stratified by TTN severity are needed to more clearly define salbutamol’s benefits.

## Supporting information

S1 Table List of excluded studies and reasons for exclusion. (PDF)

S2 Table Baseline characteristics of maternal and neonatal participants in the included studies. (PDF)

S3 Table Summary of the results of the included studies, categorized by outcome. (PDF)

S1 Fig Summary of risk of bias in the included studies assessed using the revised Cochrane risk-of-bias tool for randomized trials. (PDF)

S2 Fig Summary of risk of bias by outcome for the included studies assessed using the revised Cochrane risk-of-bias tool for randomized trials. (A) Duration of tachypnea (hours), (B) Duration of respiratory support (hours), (C) Duration of continuous positive airway pressure (CPAP) (hours), (D) Duration oxygen requirement (hours), (E) Time to initiate feeding (hours), (F) Duration of hospitalization (days), (G) Duration of neonatal intensive care unit (NICU) (days).
(PDF)

S3 Fig Funnel plots of efficacy of salbutamol in TTN infants for time to initiate feeding (hours) and duration of hospitalization (days). (A) Time to initiate feeding (hours), (B) Duration of hospitalization (days).
(PDF)

S4 Fig Sensitivity analysis for duration of respiratory support (hours), time to initiate feeding (hours), and duration of hospitalization (days). (A) Duration of respiratory support (hours), (B) Time to initiate feeding (hours), (C) Duration of hospitalization (days).
(PDF)
