# Supplementary material for: Efficacy of nebulized salbutamol in transient tachypnea of the newborn: A systematic review and meta-analysis
Source: PLoS One. 2026 Oct 1;21(10):e0359663. doi: 10.1371/journal.pone.0359663 (PMC13630241; doi:10.1371/journal.pone.0359663)
Supplement: Supplementary file 1 [file pone.0359663.s001.pdf]

S1 Table. List of excluded studies and reasons for exclusion

Author	Year	Study name	DOI	Journal	URL	Reasons for exclusion
Lin	2008	Transient tachypnea of the newborn may be an early clinical manifestation of wheezing symptoms	10.1542/peds.2008-2139ZZ	Pediatrics - Volume 122, Issue 0, pp. S205	https://www.embase.com/search/results?subaction=viewrecord&id=L354431154&from=export U2 - L354431154	Irrelevant study
EUCTR2012-001912-53	2012	Inhaled salbutamol in elective caesarean section	-	https://trialsearch.who.int/Trial2.aspx?TrialID=EUCTR2012-001912-53-FI - Volume 0, Issue 0, pp. - published 2012-01-01	https://www.cochranelibrary.com/central/doi/10.1002/central/CN-01816121/full	Registration trials
NCT01978418	2013	Inhaled Salbutamol in Elective Caesarean Section (SAISTY)	10.61186/sjku.28.6.48	https://clinicaltrials.gov/show/NCT01978418 - Volume 0, Issue 0, pp. - published 2013-01-01	https://www.cochranelibrary.com/central/doi/10.1002/central/CN-01478856/full	Irrelevant study<br>Administering salbutamol to infants delivered via cesarean section
IRCT2014062518232N1	2014	The effect of salbutamol in treatment of transient tachypnea of newborn	10.1038/s41390-020-01343-z.		https://www.cochranelibrary.com/central/doi/10.1002/central/CN-01797377/full	Registration trials
Moresco	2016	Salbutamol for transient tachypnea of the newborn	10.1002/14651858.CD011878.pub2	The Cochrane database of systematic reviews	https://pubmed.ncbi.nlm.nih.gov/27210618/	Not RCT<br>Systematic review
Keleş	2016	The Effects of Inhaled β-Adrenergic Agonists in Transient Tachypnea of the Newborn	10.1177/2333794X16645258	Global pediatric health - Volume 3, Issue , pp. 2333794X16645258	https://pubmed.ncbi.nlm.nih.gov/27336017/	Irrelevant study<br>Retrospective study
IRCT2016010225811N1	2016	Investigation of the effects of nebulized ventolin and saline .%9 in transient respiratory distress of newborn	-	https://trialsearch.who.int/Trial2.aspx?TrialID=IRCT2016010225811N1 - Volume 0, Issue 0, pp. - published	https://trialsearch.who.int/Trial2.aspx?TrialID=IRCT2016010225811N1 - Volume 0, Issue 0, pp. - published	Registration trials

Author	Year	Study name	DOI	Journal	URL	Reasons for exclusion
IRCT201711139014N201	2017	Effect of inhaler salbutamol versus placebo on treatment of neonatal transient tachypnea	-	https://trialsearch.who.int/Trial2.aspx?TrialID=IRCT201711139014N201 - Volume 0, Issue 0, pp. - published	https://www.cochranelibrary.com/central/doi/10.1002/central/CN-01897155/full	Registration trials
IRCT20170621293336N3	2017	Influence of Inhaled Salbutamol on the Outcome of Transient Tachypnea of the Newborn	-	https://trialsearch.who.int/Trial2.aspx?TrialID=IRCT2017062129336N3 - Volume 0, Issue 0, pp. - published	https://trialsearch.who.int/Trial2.aspx?TrialID=IRCT2017062129336N3 - Volume 0, Issue 0, pp. - published	Registration trials
IRCT20170814143333N80	2017	Investigation the effectiveness and non effectiveness of inhaled salbutamol in the treatment of respiratory diseases	-	https://trialsearch.who.int/Trial2.aspx?TrialID=IRCT2017081414333N80 - Volume 0, Issue 0, pp. - published 2017-01-01	https://www.cochranelibrary.com/central/doi/10.1002/central/CN-01894137/full	Registration trials
Taniguchi	2018	Inhaled procaterol for the treatment of transient tachypnea of the newborn	10.1111/ped.13699		https://pubmed.ncbi.nlm.nih.gov/30246320/	Irrelevant study<br>Inhaled procaterol
Çelik	2018	Does inhaled salbutamol before surfactant therapy have any beneficial effect?	10.24953/turkjped.2018.06.007	The Turkish journal of pediatrics - Volume 60, Issue 6, pp. 669-674 - published 2018-01-01	https://pubmed.ncbi.nlm.nih.gov/31365203/	Irrelevant study<br>Inhaled salbutamol or normal saline was administered prior to the first dose of surfactant
IRCT20190328043133N1 Abolhasan Choobdar	2019	The effect of inhaled salbutamol on treatment of Transient tachypnoea of newborn	-	https://trialsearch.who.int/Trial2.aspx?TrialID=IRCT20190328043133N1 - Volume 0, Issue 0, pp. - published 2023-01-01	https://www.cochranelibrary.com/central/doi/10.1002/central/CN-02540943/full	Registration trials
Iqbal	2020	Efficacy of salbutamol nebulization for the	-	Pak. Paediatr. J. - Volume 44, Issue 4, pp. 28-29	https://www.embase.com/search/results?subaction=viewrecord&id=	Abstract conference

Author	Year	Study name	DOI	Journal	URL	Reasons for exclusion
		treatment of transient tachypnea of newborn			L634251177&from=export U2 - L634251177	
IRCT20170201032346N4	2020	The effects of nebulized salbutamol on transient tachypnea of neonates treatment	-	https://trialsearch.who.int/Trial2.aspx?TrialID=IRCT20170201032346N4 - Volume 0, Issue 0, pp. - published 2020-01-01	https://trialsearch.who.int/Trial2.aspx?TrialID=IRCT20170201032346N4	Registration trials
Moresco	2021	Salbutamol for transient tachypnea of the newborn	10.1002/14651858.CD011878.pub3	The Cochrane database of systematic reviews - Volume 2, Issue 2, pp. CD011878 - published 2021-02-05	https://pubmed.ncbi.nlm.nih.gov/33543473/	Not RCT<br>Systematic review
NCT05006235	2021	Inhaled Beta-2 Agonist Versus Epinephrine For Treatment of Transient Tachypnea of Newborn (TTN)	-	https://clinicaltrials.gov/show/NCT05006235 - Volume 0, Issue 0, pp. - published 2021-01-01	https://clinicaltrials.gov/study/NCT05006235?cond=NCT05006235&rank=1	Registration trials
Madajczak	2022	Protocol for a multicenter, double-blind, randomized, placebo-controlled phase III trial of the inhaled β2-adrenergic receptor agonist salbutamol for transient tachypnea of the newborn (the REFSAL trial)	10.3389/fped.2022.1060843	Frontiers in pediatrics - Volume 10, Issue , pp. 1060843	https://pubmed.ncbi.nlm.nih.gov/36714639/	Prtotocol
NCT05527704	2022	The Inhaled β2-adrenergic Receptor Agonist for Transient Tachypnoea of the Newborn (the REFSAL Trial)	-	https://clinicaltrials.gov/ct2/show/NCT05527704 - Volume 0, Issue 0, pp. - published 2022-01-01	https://www.cochranelibrary.com/central/doi/10.1002/central/CN-02455447/full	Registration trials

Author	Year	Study name	DOI	Journal	URL	Reasons for exclusion
IRCT20091201002801N6	2023	Comparison of Ventolin nebulizer with supportive treatment in Transient tachypnea of the newborns (TTN) admitted to the neonatal intensive care unit (NICU) of Imam Khomeini and Bouali Sari hospitals of Sari	-	https://trialsearch.who.int/Trial2.aspx?TrialID=IRCT20091201002801N6 - Volume 0, Issue 0, pp. - published 2023-01-01	https://www.cochranelibrary.com/central/doi/10.1002/central/CN-02586226/full	Registration trials
IRCT20221028056324N1	2023	Effect of nebulized Salbutamol versus Epinephrine in treatment of transient tachypnea of neonates	-	https://trialsearch.who.int/Trial2.aspx?TrialID=IRCT20221028056324N1 - Volume 0, Issue 0, pp. - published 2023-01-01	https://www.cochranelibrary.com/central/doi/10.1002/central/CN-02604916/full	Registration trials
CTRI/2023/05/052441	2023	Use of salbutamol mist to treat breathing problem in babies soon after birth	-	https://trialsearch.who.int/Trial2.aspx?TrialID=CTRI/2023/05/052441 - Volume 0, Issue 0, pp. - published 2023-01-01	https://www.cochranelibrary.com/central/doi/10.1002/central/CN-02565859/full	Registration trials
ACTRN12624000987561	2024	Breathing in Newborns: evaluating the Impact of Salbutamol, Furosemide, and Their Combination on Newborn Breathing at CMH Peshawar's Neonatal Intensive Care Unit	-	https://trialsearch.who.int/Trial2.aspx?TrialID=ACTRN12624000987561 - Volume 0, Issue 0, pp. - published 2024-01-01	https://www.cochranelibrary.com/central/doi/10.1002/central/CN-02740800/full	Registration trials
CTRI/2025/11/097695	2025	Effect of Nebulized Salbutamol in time taken to recover in Transient Tachypnea of newborn	-	-	https://www.cochranelibrary.com/central/doi/10.1002/central/CN-02942892/full	Registration trials
IRCT20250811066816N1	2025	Effect of salbutamol on neonatal transient tachypnea	-	-	https://www.cochranelibrary.com/central/doi/10.1002/central/CN-02908919/full	Registration trials

Author	Year	Study name	DOI	Journal	URL	Reasons for exclusion
IRCT20250814066854N1	2025	The Effect of Inhaled Salbutamol on Respiratory Improvement in Neonates with Transient Tachypnea of the Newborn (TTN)	-	-	https://www.cochranelibrary.com/central/doi/10.1002/central/CN-02943780/full	Registration trials
PACTR202506603201609	2025	The effect of inhaled salbutamol and ipratropium bromide on improving the outcomes of transient tachypnea of newborn: clinical trial	-	-	https://www.cochranelibrary.com/central/doi/10.1002/central/CN-02877746/full	Registration trials
Priyadarshi	2026	Effect of Nebulized Salbutamol on Lung Ultrasound Aeration Score in Transient Tachypnea of Newborn	10.1007/s12098-025-05952-6	Indian J. Pediatr. - Volume 93, Issue 3, pp. 309 - published 2026-01-01	https://www.embase.com/search/results?subaction=viewrecord&id=L2042290529&from=export U2 - L2042290529	Irrelevant study Outcomes: Lung Ultrasound Aeration Score
-	2026	The Effectiveness of Inhaled Salbutamol in the Management of Transient Tachypnea of the Newborn(TTN)	-	clinicaltrials.gov - Volume 0, Issue 0, pp. - published 2026-01-01	https://www.embase.com/search/results?subaction=viewrecord&id=LNCT07350421&from=export U2 - LNCT07350421 U3 - 2026-04-21 U4 - 2026-04-21	Registration trials

RCT, randomized controlled trial.
