# Supplementary material for: Efficacy of nebulized salbutamol in transient tachypnea of the newborn: A systematic review and meta-analysis
Source: PLoS One. 2026 Oct 1;21(10):e0359663. doi: 10.1371/journal.pone.0359663 (PMC13630241; doi:10.1371/journal.pone.0359663)
Supplement: Supplementary file 2 [file pone.0359663.s002.pdf]

## S2 Table. Baseline maternal and neonatal characteristics of the participants in the included studies

| Author | Year | Number of participants (N) | | GA (wks) mean ± SD | | Birth weight (g) mean ± SD | | Sex Male/Female (N) | | Mean TTN clinical score (baseline) | |
|---|---|---|---|---|---|---|---|---|---|---|---|
| | | I | C | I | C | I | C | I | C | I | C |
| Armangil | 2011 | 32 | 22 | 37.0 ± 1.6 | 36.7 ± 1.6 | 2991 ± 536 | 2990 ± 574 | 21/11 | 17/5 | 8.0 ± 1.5* | 7.0 ± 0.7* |
| Kim | 2014 | 28 | 12 | 37.6 ± 1.9 | 37.5 ± 1.0 | 3091 ± 591 | 3203 ± 432 | 17/11 | 7/5 | NR | |
| Monzoy-Ventre | 2015 | 15 | 15 | 36.6 ± 1.7 | 36.6 ± 2.5 | 2455 ± 703 | 2519 ± 592 | 8/7 | 9/6 | NR | |
| Kaur | 2017 | 50 | 50 | 38.4 ± 1.2 | 38.5 ± 1.3 | 3800 ± 1770 | 3500 ± 1770 | 65/35 | | 5.7 ± 1.0 | 5.6 ± 1.1 |
| Mohammadzadeh | 2017 | 35 | 35 | 36.9 ± 2 | 36.7 ± 1.7 | 2988 ± 720 | 2988 ± 720 | 22/13 | 23/12 | 2.5 ± 1.0 | 2.4 ± 1.3 |
| Mussavi | 2017 | 30 | 30 | 37.0 ± 1.7 | 36.9 ± 1.7 | 2676 ± 611 | 2731 ± 576 | 13/17 | 18/12 | NR | |
| Malakian | 2018 | 74 | 74 | 37.6 ± 1.5 | 37.4 ± 1.9 | 2810 ± 440 | 2810 ± 500 | 54/20 | 47/27 | 8.4 ± 1.8 | 8.8 ± 2.1 |
| Babaei | 2019 | 40 | 40 | 36.7 ± 1.1 | 36.3 ± 1.4 | 3181 ± 697 | 3069 ± 577 | 24 /16 | 29/11 | 7.9 ± 0.7 | 7.4± 1.0 |
| Al Lahony | 2020 | 30 | 30 | 38 ± 0.2* | 38 ± 1.5* | 3320 ± 240* | 3280 ± 410* | 13/17 | 17/13 | 8.0 ± 0.7* | 8.0 ± 0.7* |
| Salama | 2020 | 50 | 50 | 38.5 ± 0.9 | 38.4 ± 0.8 | 3330 ± 420 | 3340 ± 390 | 24/26 | 26/24 | 7.7 ± 1.0 | 7.7 ± 0.9 |
| Talaat | 2020 | 54 | 46 | 36.7 ± 1.7 | 36.9 ± 1.6 | 2826 ± 415 | 2883 ± 438 | 32/22 | 27/19 | 8.0 ± 2.2* | 7.0 ± 2.2* |
| El-Badawy | 2021 | 25 | 25 | 37.5 ± 0.7 | 37.7± 1.0 | 3620 ± 600 | 3550 ± 670 | 16/9 | 17/8 | 2.5 ± 0. | 2.9 ± 1.0 |
| Maroof | 2021 | 50 | 50 | 39.9 ± 1.4 | 40.0 ± 1.5 | 2963 ± 283 | 3035 ± 274 | 26/24 | 26/24 | NR | |
| Ahmed | 2022 | 30 | 30 | 38.4 ±1.4 | 38.3±1.2 | 2970± 250 | 2990 ± 270 | 28/32 | | 8.3 ± 1.4 | 8.0 ± 1.2 |
| Basiri | 2022 | 26 | 26 | 36.3 ± 1.9 | 36.6 ± 1.5 | 2874 ± 580 | 2772 ± 470 | 12/14 | 15/11 | 2.1 ± 0.9 | 2.0 ± 0.7 |
| Hamed | 2022 | 50 | 50 | >36 weeks | | 3040 ± 640 | 3080 ± 680 | 26/24 | 28/22 | NR | |
| Khushdil | 2022 | 25 | 25 | 38.8 ± 6.2 | | 2558 ± 620 | | 48/52 | | 4.6 ± 1.4 | |
| Sabry | 2022 | 25 | 50 | 37.8 ± 0.9 | 37.5 ± 0.9 | 3094 ± 193 | 2965 ± 440 | 15/10 | 30/20 | 9.1 ± 0.9 | 8.5 ± 1.1 |
| Anwar | 2023 | 40 | 40 | 34 - 41weeks | | 2470 ± 540 | 2460 ± 590 | 23/17 | 22/18 | 7.0 ± 1.0 | 7.2 ± 1.0 |
| Choobdar | 2024 | 30 | 30 | 36.3 ± 1.1 | 36.3 ± 1.0 | 2880 ± 538 | 2816 ± 484 | NR | | 5.3 ± 1.6 | 5.3 ± 1.7 |
| Dhaka | 2024 | 67 | 67 | 37.0 ± 2.0 | 36.7 ± 1.9 | 2769 ± 579 | 2610 ± 530 | 40/27 | 41/26 | 2.0 ± 0.7* | 2.0 ± 0.7* |
| Alvi | 2025 | 30 | 30 | 37.7 ± 1.0 | 37.9 ± 1.2 | 3115 ± 134 | 3132 ± 129 | 17/13 | 16/14 | NR | |
| Gooran | 2025 | 32 | 32 | 37.5 ± 1.0 | 36.8 ± 1.8 | 3139 ± 521 | 3071 ± 689 | 27/5 | 22/5 | 6.3 ± 1.7 | 5.8 ± 1.7 |
| Hussain | 2025 | 30 | 30 | 38.5 ± 1.2 | 38.4 ± 1.1 | 3200 ± 300 | 3100 ± 200 | 18/12 | 17/13 | 6.5 ± 0.5 | 6.4 ± 0.4 |

| Author | Year | Number of participants (N) | | GA (wks) mean ± SD | | Birth weight (g) mean ± SD | | Sex Male/Female (N) | | Mean TTN clinical score (baseline) | |
|---|---|---|---|---|---|---|---|---|---|---|---|
| | | I | C | I | C | I | C | I | C | I | C |
| Ullah | 2025 | 30 | 30 | 39.3 ± 1.3 | 38.9 ± 1.2 | 2871 ± 280 | 2779 ± 230 | 14/16 | 20/10 | NR | |

The results presented as mean ± SD
* Converted data from Median (IQR 1-3)  to mean (SD)
C, Control; g, gram; GA, Gestational age; I, intervention; NR, No report; SD, standard deviation
