# Supplementary material for: Efficacy of nebulized salbutamol in transient tachypnea of the newborn: A systematic review and meta-analysis
Source: PLoS One. 2026 Oct 1;21(10):e0359663. doi: 10.1371/journal.pone.0359663 (PMC13630241; doi:10.1371/journal.pone.0359663)
Supplement: Supplementary file 3 [file pone.0359663.s003.pdf]

S3 Table. Summary of the results of the included studies, categorized by outcome

Author (year)	Salbutamol			NSS or standard of care			Mean difference (95% CI)
	Mean	SD	N	Mean	SD	N	
**Respiratory outcomes**							
**Duration of tachypnea (hours)**							
Kim 2014	31.3	23.7	28	53.5	56.8	12	-22.20 [-55.51, 11.11]
Babaei 2019	34.45	8.74	40	51.12	16.09	40	-16.67 [-22.34, -11.00]
Dhaka 2024*	12.5	4.44	67	12.0	2.67	67	0.50 [-0.74, 1.74]
Total (95% CI)			135			119	-9.93 [-25.42, 5.56]
Heterogeneity: $Tau^2 = 141.80$; $Chi^2 = 35.23$, df = 2 ($P < 0.00001$); $I^2 = 94\%$							
Test for overall effect: Z = 1.26 (P = 0.21)							
**Duration of respiratory support (hours)**							
Armangil 2011*	30.0	44.44	32	48.0	53.33	22	-18.00 [-45.09, 9.09]
Talaat 2020	49.17	15.86	54	61.43	16.81	46	-12.26 [-18.70, -5.82]
El-Badawy 2021	21.52	8.17	25	38.12	14.32	25	-16.60 [-23.06, -10.14]
Basiri 2022	58.08	16.8	26	75.6	19.92	26	-17.52 [-27.54, -7.50]
Dhaka 2024*	12.0	4.44	67	12.0	2.96	67	0.00 [-1.28, 1.28]
Gooran 2025	34.56	25.854	32	67.5	36.976	32	-32.94 [-48.57, -17.31]
Total (95% CI)			236			218	-14.85 [-25.05, -4.65]
Heterogeneity: $Tau^2 = 128.48$; $Chi^2 = 63.77$, df = 5 ($P < 0.00001$); $I^2 = 92\%$							
Test for overall effect: Z = 2.85 (P = 0.004)							
**Duration of Continuous Positive Airway Pressure (CPAP) (hours)**							
Mussavi 2017	38.4	18.48	30	79.2	23.52	30	-40.80 [-51.50, -30.10]
Malakian 2018	11.41	12.2	74	21.68	22.82	74	-10.27 [-16.17, -4.37]
Babaei 2019	23.92	18.15	40	30.5	24.49	40	-6.58 [-16.03, 2.87]
Sabry 2022	24.17	11.75	25	41.16	15.95	50	-16.99 [-23.37, -10.61]
Total (95% CI)			169			194	-18.19 [-30.03, -6.35]
Heterogeneity: $Tau^2 = 128.30$; $Chi^2 = 28.12$, df = 3 ($P < 0.00001$); $I^2 = 89\%$							

Author (year)	Salbutamol			NSS or standard of care			Mean difference (95% CI)
	Mean	SD	N	Mean	SD	N	
Test for overall effect: Z = 3.01 (P = 0.003)							
**Duration of oxygen supplementation (hours)**							
Kim 2014	34.2	32.2	25	77.3	64.7	12	-43.10 [-81.82, -4.38]
Mohammadzadeh 2017	18.7	12.5	35	26.0	29.3	35	-7.30 [-17.85, 3.25]
Malakian 2018	30.36	19.52	74	58.92	33.87	74	-28.56 [-37.47, -19.65]
Babaei 2019	41.8	7.77	40	60.05	18.15	40	-18.25 [-24.37, -12.13]
Hamed 2022	23.42	6.71	46	67.4	12.69	30	-43.98 [-48.92, -39.04]
Khusdil 2022	58.0	53.5	25	83.64	68.54	25	-25.64 [-59.72, 8.44]
Sabry 2022	61.12	19.31	25	85.32	24.35	50	-24.20 [-34.34, -14.06]
Anwar 2023	25.6	10.98	40	42.63	8.87	40	-17.03 [-21.40, -12.66]
Total (95% CI)			310			306	-24.57 [-35.03, -14.10]
Heterogeneity: $Tau^2 = 176.40$; $Chi^2 = 86.34$, df = 7 ($P < 0.00001$); $I^2 = 92\%$							
Test for overall effect: Z = 4.60 (P < 0.00001)							
**Time to initiate feeding (hours)**							
Kim 2014	12.1	19.9	28	28.5	45.9	12	-16.40 [-43.40, 10.60]
Mohammadzadeh 2017	28.9	10.1	35	36.8	27.8	35	-7.90 [-17.70, 1.90]
Malakian 2018	36.92	19.08	74	67.18	41.12	74	-30.26 [-40.59, -19.93]
Babaei 2019	53.28	10.08	40	85.2	78.72	40	-31.92 [-56.51, -7.33]
Salama 2020	26.72	3.68	50	39.66	4.7	50	-12.94 [-14.59, -11.29]
Hamed 2022	26.6	3.72	50	32.74	4.28	50	-6.14 [-7.71, -4.57]
Sabry 2022	30.16	11.73	25	32.24	13.02	50	-2.08 [-7.93, 3.77]
Basiri 2022	56.4	11.52	26	54.48	10.8	26	1.92 [-4.15, 7.99]
Choobdar 2024	10.17	2.42	30	16.57	5.66	30	-6.40 [-8.60, -4.20]
Dhaka 2024*	1.0	0.37	67	1.0	0.37	67	0.00 [-0.13, 0.13]
Gooran 2025	66.0	21.12	32	63.12	14.616	32	2.88 [-6.02, 11.78]
Total (95% CI)			457			466	-7.16 [-11.77, -2.55]
Heterogeneity: $Tau^2 = 43.84$; $Chi^2 = 365.89$, df = 10 ($P < 0.00001$); $I^2 = 97\%$							

Author (year)	Salbutamol			NSS or standard of care			Mean difference (95% CI)
	Mean	SD	N	Mean	SD	N	
Test for overall effect: Z = 3.05 (P = 0.002)							
**Duration of hospitalization (days)**							
Armangil 2011*	4.0	2.22	32	6.0	2.22	22	-2.00 [-3.21, -0.79]
Kim 2014	8.5	3.9	28	8.8	3.2	12	-0.30 [-2.62, 2.02]
Mohammadzadeh 2017	4.8	1.2	35	5.4	2.4	35	-0.60 [-1.49, 0.29]
Malakian 2018	3.94	1.32	74	5.66	2.32	74	-1.72 [-2.33, -1.11]
Babaei 2019	4.92	0.82	40	6.52	1.06	40	-1.60 [-2.02, -1.18]
Talaat 2020	3.89	1.08	54	4.76	1.14	46	-0.87 [-1.31, -0.43]
Salama 2020	3.78	1.25	50	7.36	1.28	50	-3.58 [-4.08, -3.08]
El-Badawy 2021	2.0	0.38	25	2.56	0.46	25	-0.56 [-0.79, -0.33]
Hamed 2022	3.12	0.43	50	5.08	1.14	50	-1.96 [-2.30, -1.62]
Basiri 2022	6.31	1.05	26	5.77	1.11	26	0.54 [-0.05, 1.13]
Anwar 2023	1.8	0.68	40	2.68	0.42	40	-0.88 [-1.13, -0.63]
Choobdar 2024	2.55	0.66	30	2.73	0.58	30	-0.18 [-0.49, 0.13]
Gooran 2025	4.69	1.148	32	5.88	1.008	32	-1.19 [-1.72, -0.66]
Total (95% CI)			516			482	-1.17 [-1.69, -0.65]
Heterogeneity: Tau² = 0.79; Chi² = 219.94, df = 12 (P < 0.00001); I² = 95%							
Test for overall effect: Z = 4.43 (P < 0.00001)							
**Duration of neonatal intensive care unit stay (days)**							
Sabry 2022	3.32	0.91	25	5.09	1.36	50	-1.77 [-2.29, -1.25]
Dhaka 2024*	0.88	0.23	67	0.83	0.18	67	0.05 [-0.02, 0.12]
Heterogeneity: Tau² = 1.62; Chi² = 46.40, df = 1 (P < 0.00001); I² = 98%							
Test for overall effect: Z = 0.92 (P = 0.36)							

* Converted data from Median (IQR 1-3) to mean (SD)
CI, confidence interval; FiO2, fraction of inspired oxygen; SD, standard Deviation; TTN, transient tachypnea of the Newborn.
