# Supplementary material for: Efficacy of nebulized salbutamol in transient tachypnea of the newborn: A systematic review and meta-analysis
Source: PLoS One. 2026 Oct 1;21(10):e0359663. doi: 10.1371/journal.pone.0359663 (PMC13630241; doi:10.1371/journal.pone.0359663)
Supplement: Supplementary file 4 [file pone.0359663.s004.pdf]

S1 Fig. Summary of risk of bias in the included studies assessed using the revised Cochrane risk-of-bias tool for randomized trials

Risk of bias domains

Study	D1	D2	D3	D4	D5	Overall
Armangil 2011	-	+	+	+	+	-
Kim 2014	-	+	+	-	+	-
Monzoy-Ventre 2015	-	+	+	-	+	-
Kaur 2017	-	+	+	-	+	-
Mohammadzadeh 2017	-	+	+	-	+	-
Mussavi 2017	-	+	+	-	+	-
Malakian 2018	-	+	+	+	+	-
Babaei 2019	-	+	+	-	+	-
Al Lahony 2020	-	+	+	-	+	-
Salama 2020	-	+	+	-	+	-
Talaat 2020	-	+	+	-	+	-
El-Badawy 2021	-	+	+	-	+	-
Maroof 2021	-	+	+	-	+	-
Ahmed 2022	-	+	+	-	+	-
Basiri 2022	+	+	+	-	+	-
Hamed 2022	-	+	+	-	+	-
Khushdil 2022	-	+	+	-	+	-
Sabry 2022	+	+	+	-	+	-
Anwar 2023	-	+	+	-	+	-
Choobdar 2024	+	+	+	+	+	+
Dhaka 2024	+	+	+	+	+	+
Alvi 2025	-	+	+	-	+	-
Gooran 2025	+	+	+	-	+	-
Hussain 2025	-	+	+	-	+	-
Ullah 2025	-	+	+	-	+	-

Domains:
D1: Bias arising from the randomization process.
D2: Bias due to deviations from intended intervention.
D3: Bias due to missing outcome data.
D4: Bias in measurement of the outcome.
D5: Bias in selection of the reported result.

Judgement
- Some concerns
+ Low
