# Supplementary material for: Efficacy of nebulized salbutamol in transient tachypnea of the newborn: A systematic review and meta-analysis
Source: PLoS One. 2026 Oct 1;21(10):e0359663. doi: 10.1371/journal.pone.0359663 (PMC13630241; doi:10.1371/journal.pone.0359663)

S2 Fig. Summary of risk of bias by outcome for the included studies assessed using the revised Cochrane risk-of-bias tool for randomized trials.

(A) Duration of tachypnea (hours)

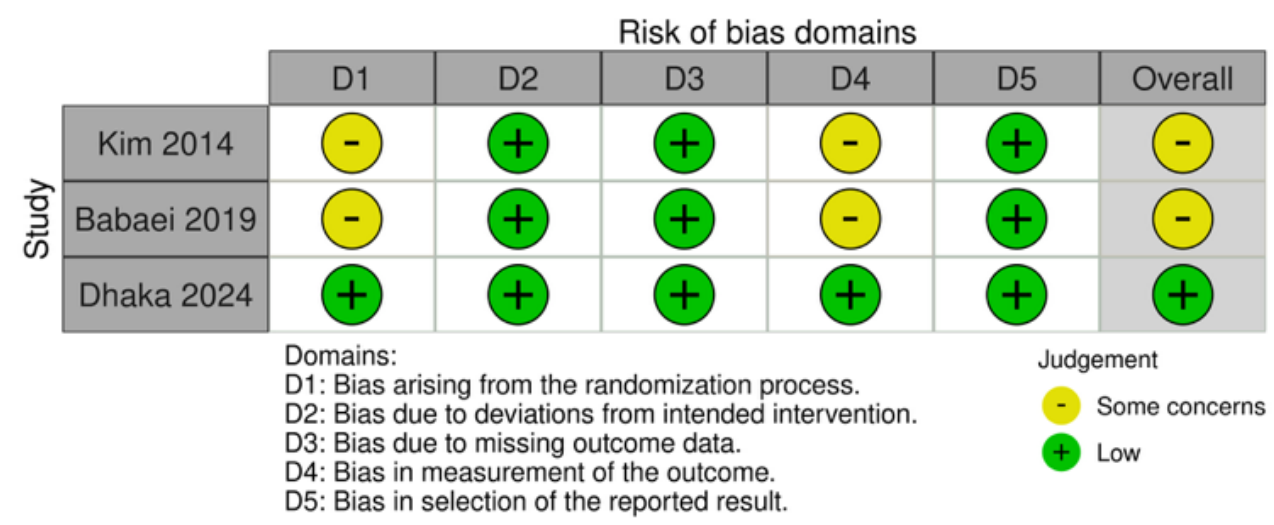

(B) Duration of respiratory support (hours)

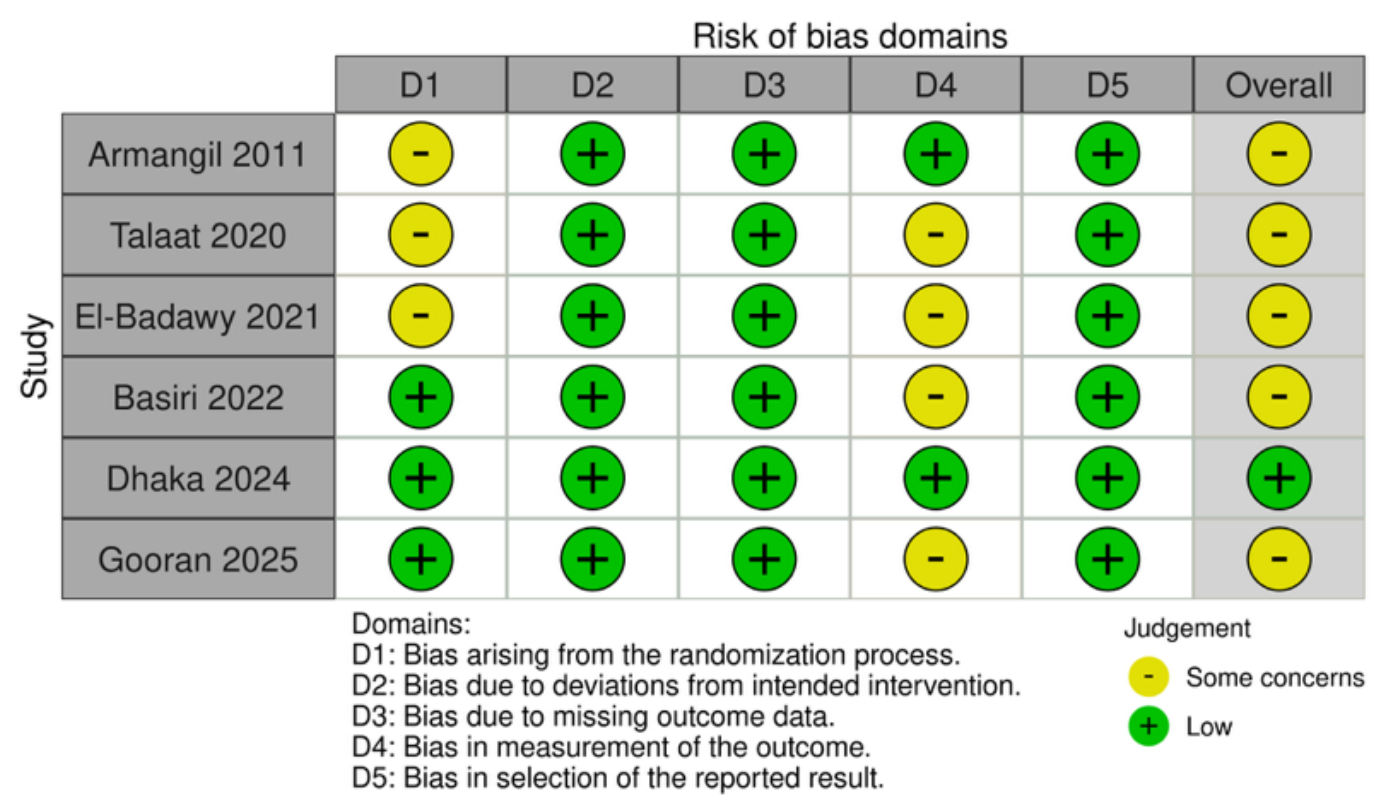

(C) Duration of continuous positive airway pressure (CPAP) (hours)

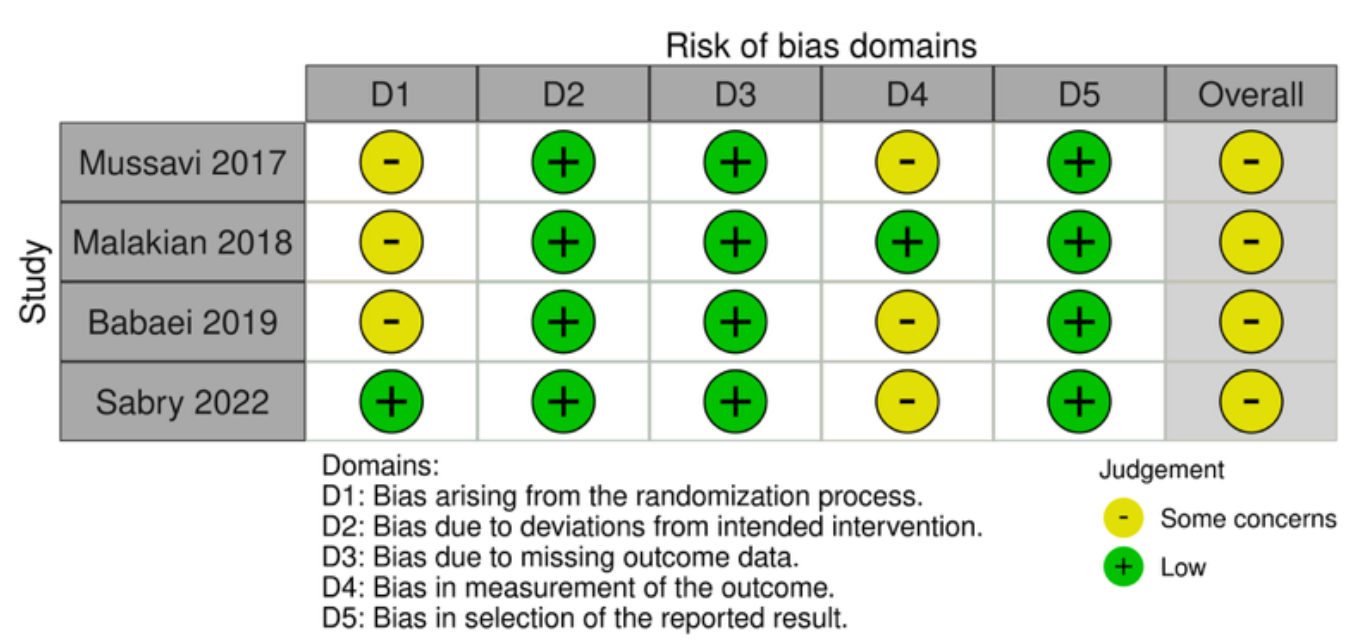

(D) Duration oxygen requirement (hours)

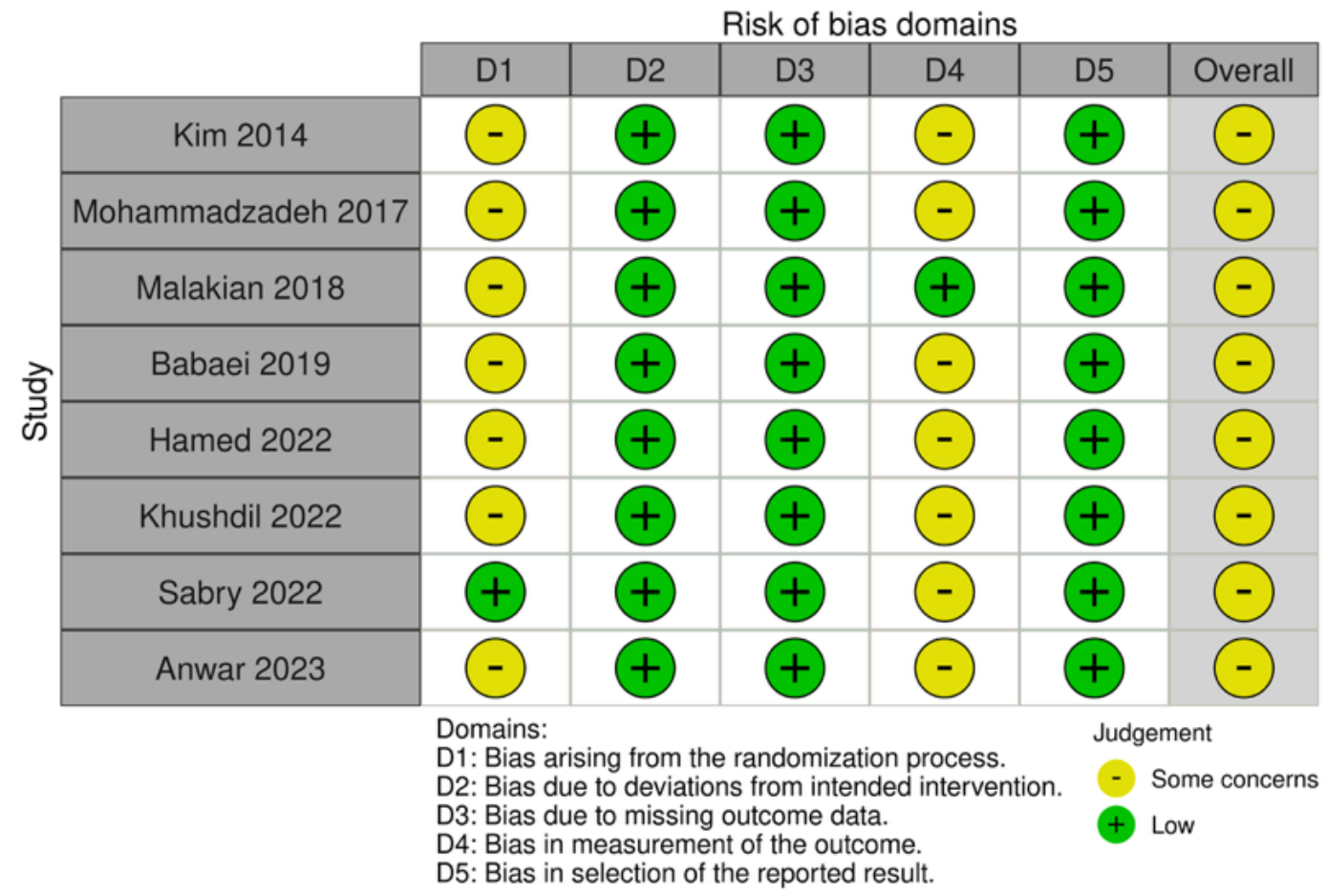

(E) Time to initiate feeding (hours)

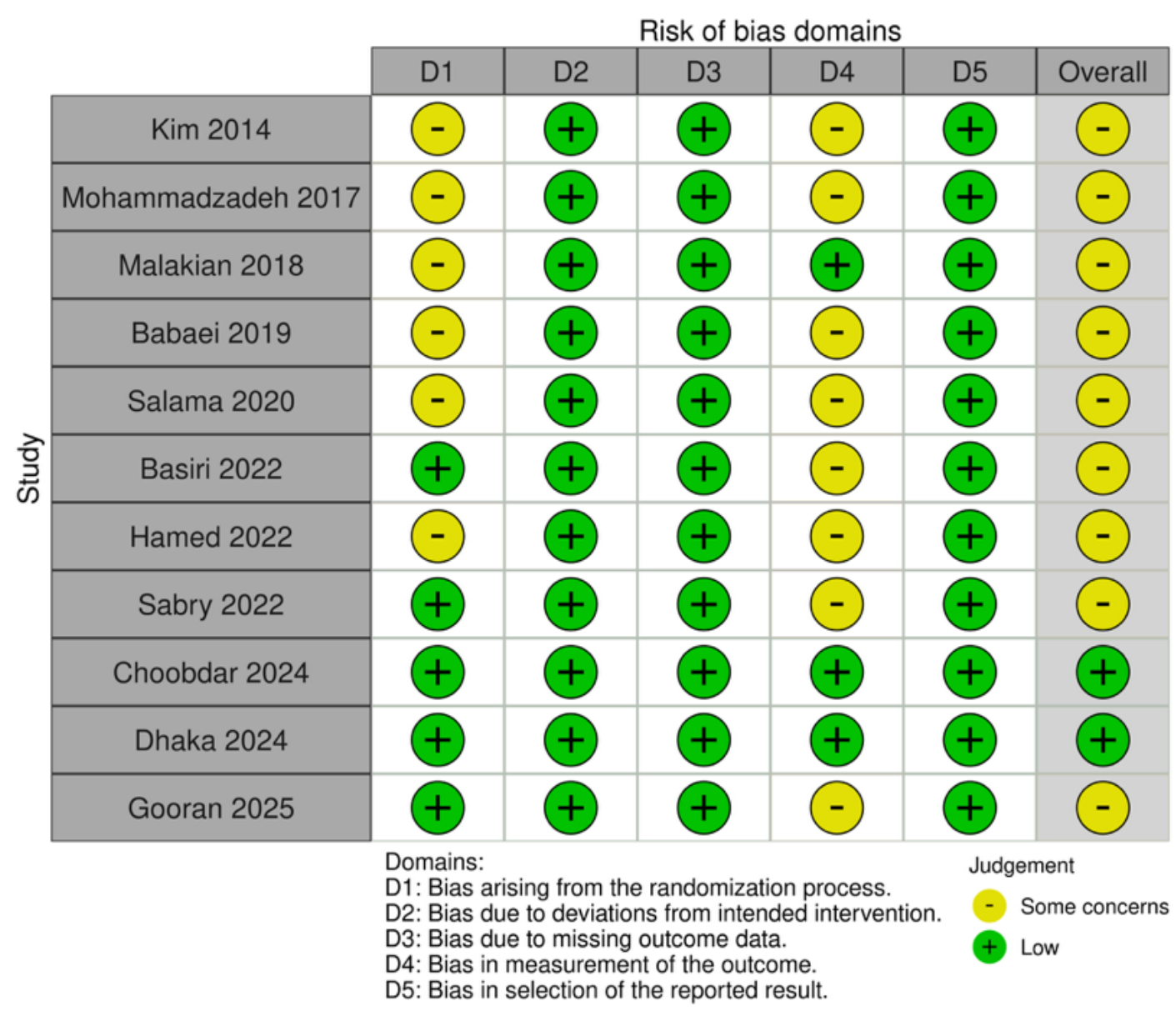

(F) Duration of hospitalization (days)

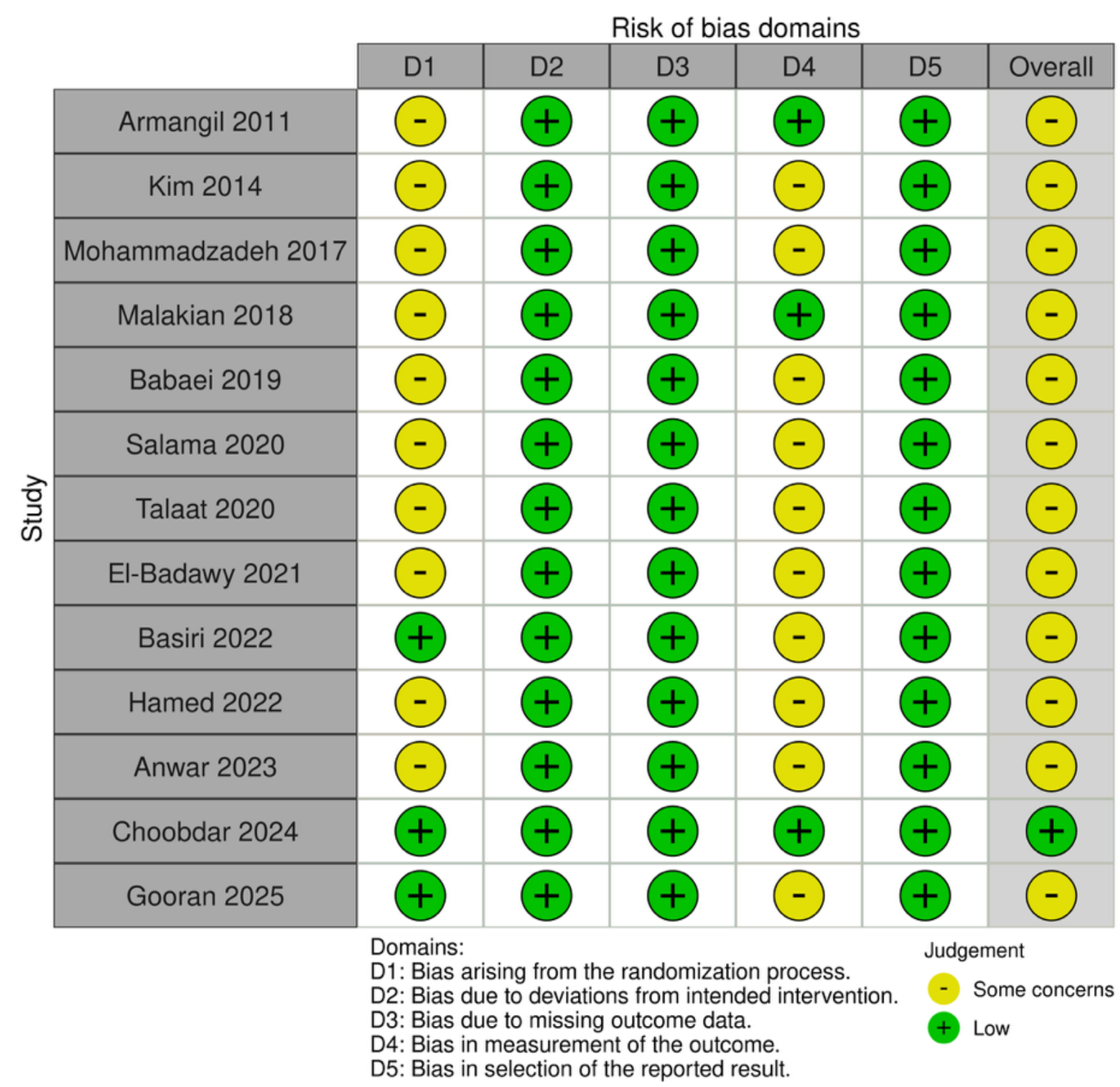

(G) Duration of neonatal intensive care unit (NICU) (days)

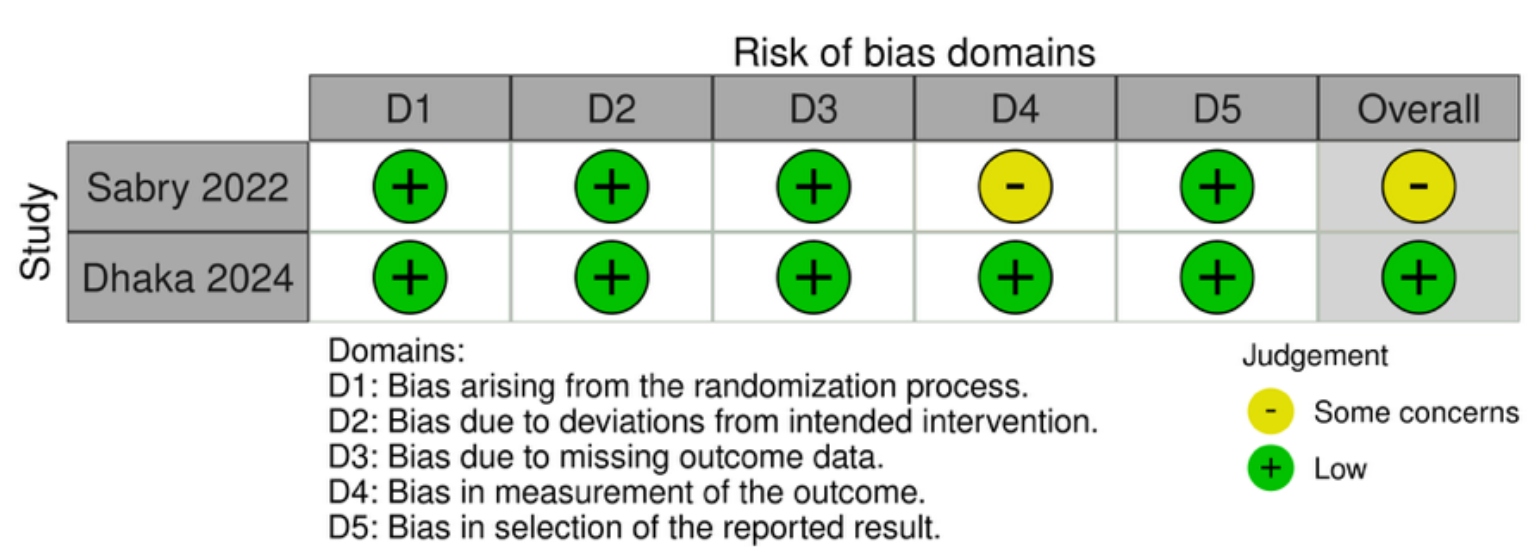

Supplement: Supplementary file 5 [file pone.0359663.s005.pdf]
