# Supplementary material for: Efficacy of nebulized salbutamol in transient tachypnea of the newborn: A systematic review and meta-analysis
Source: PLoS One. 2026 Oct 1;21(10):e0359663. doi: 10.1371/journal.pone.0359663 (PMC13630241; doi:10.1371/journal.pone.0359663)

S3 Fig. Funnel plots of efficacy of salbutamol in TTN infants for time to initiate feeding (hours) and duration of hospitalization (days).

(A) Time to initiate feeding (hours)

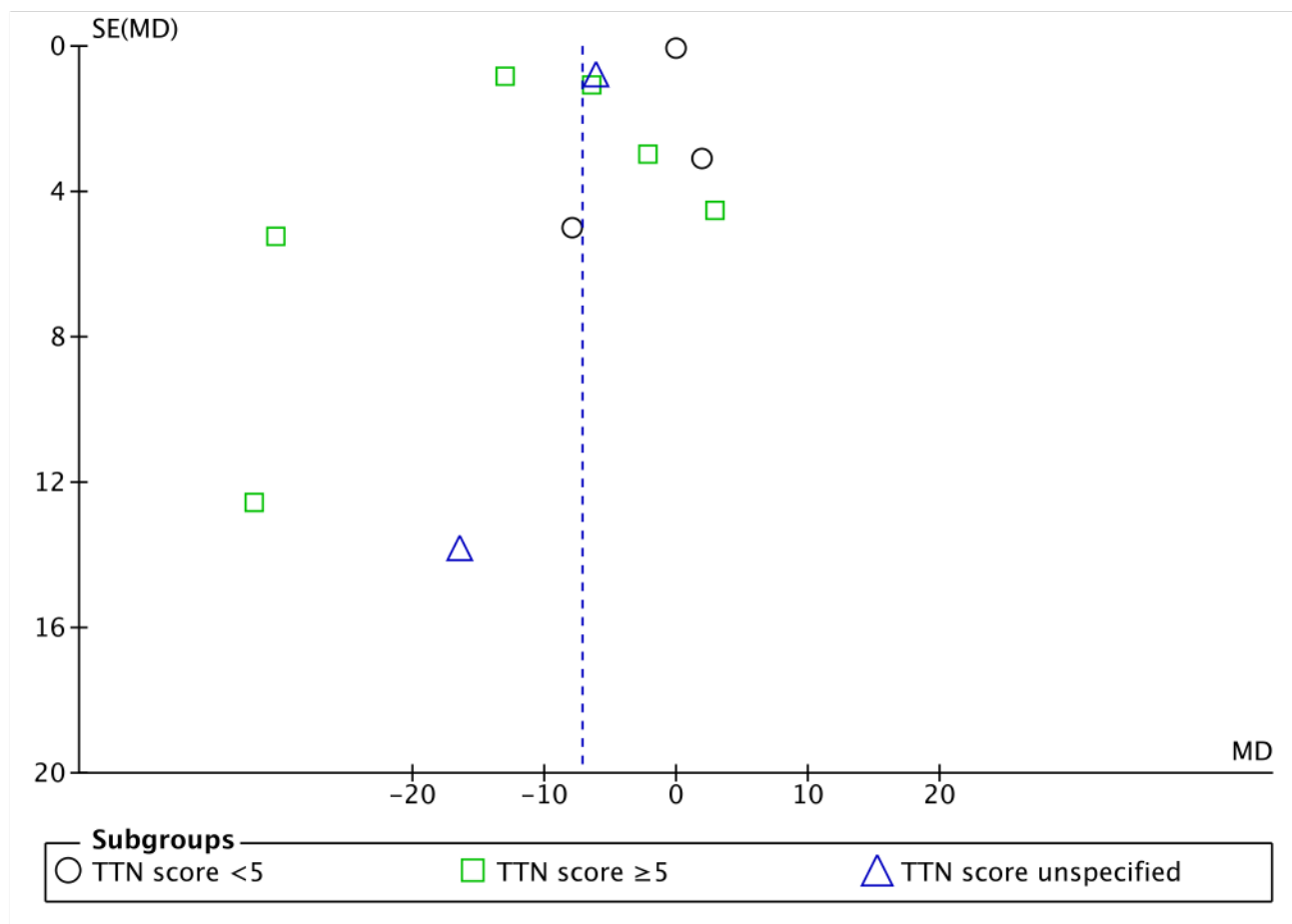

(B) Duration of hospitalization (days)

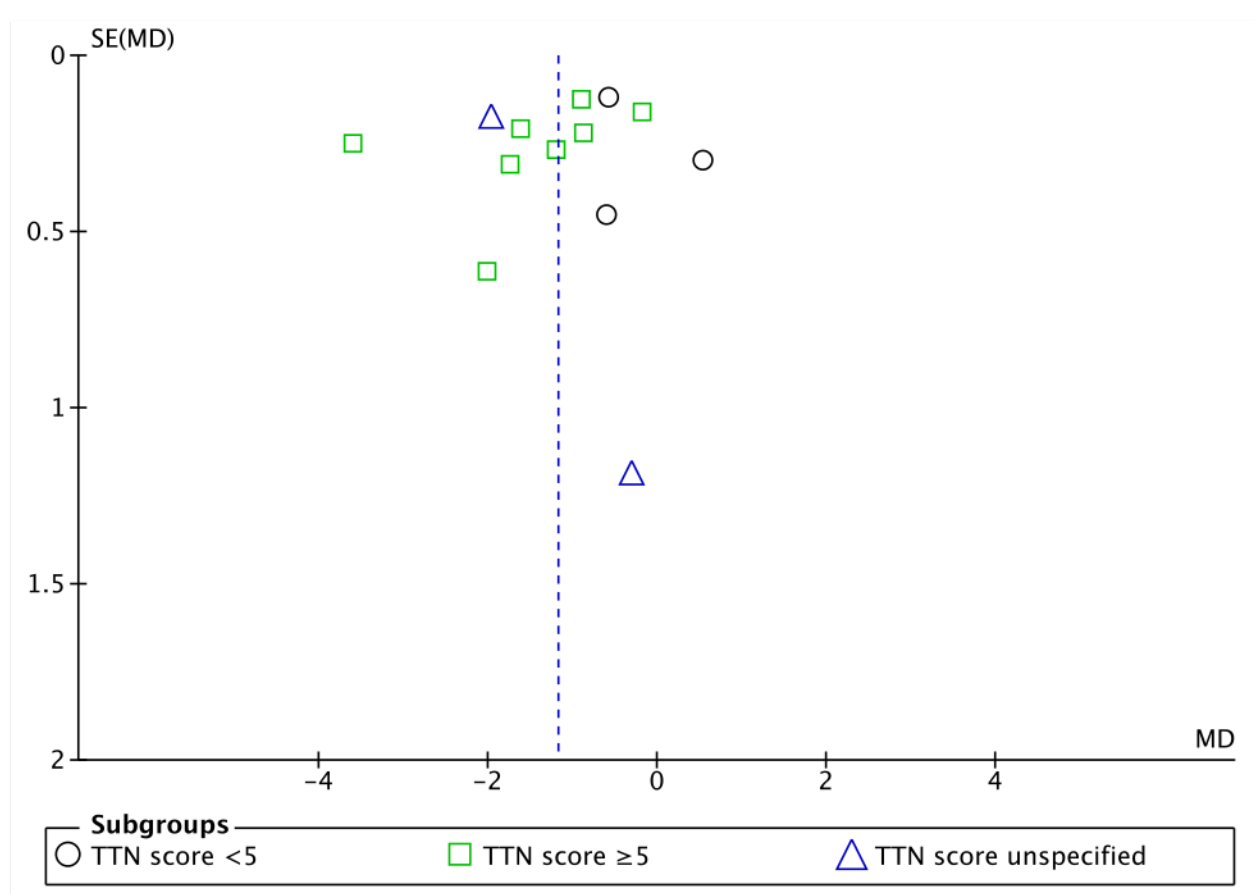

Supplement: Supplementary file 6 [file pone.0359663.s006.pdf]
