# Supplementary material for: Efficacy of nebulized salbutamol in transient tachypnea of the newborn: A systematic review and meta-analysis
Source: PLoS One. 2026 Oct 1;21(10):e0359663. doi: 10.1371/journal.pone.0359663 (PMC13630241; doi:10.1371/journal.pone.0359663)

S4 Fig. Sensitivity analysis for duration of respiratory support (hours), time to initiate feeding (hours), and duration of hospitalization (days)

(A) Duration of respiratory support (hours)

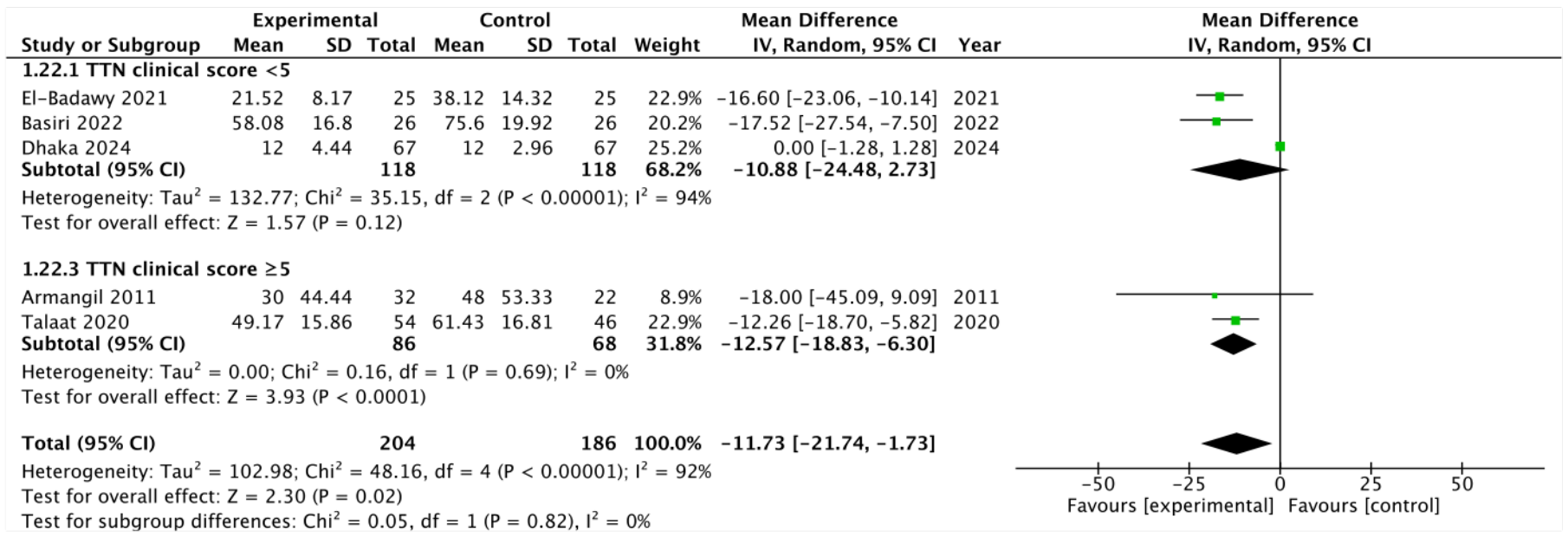

(B) Time to initiate feeding (hours)

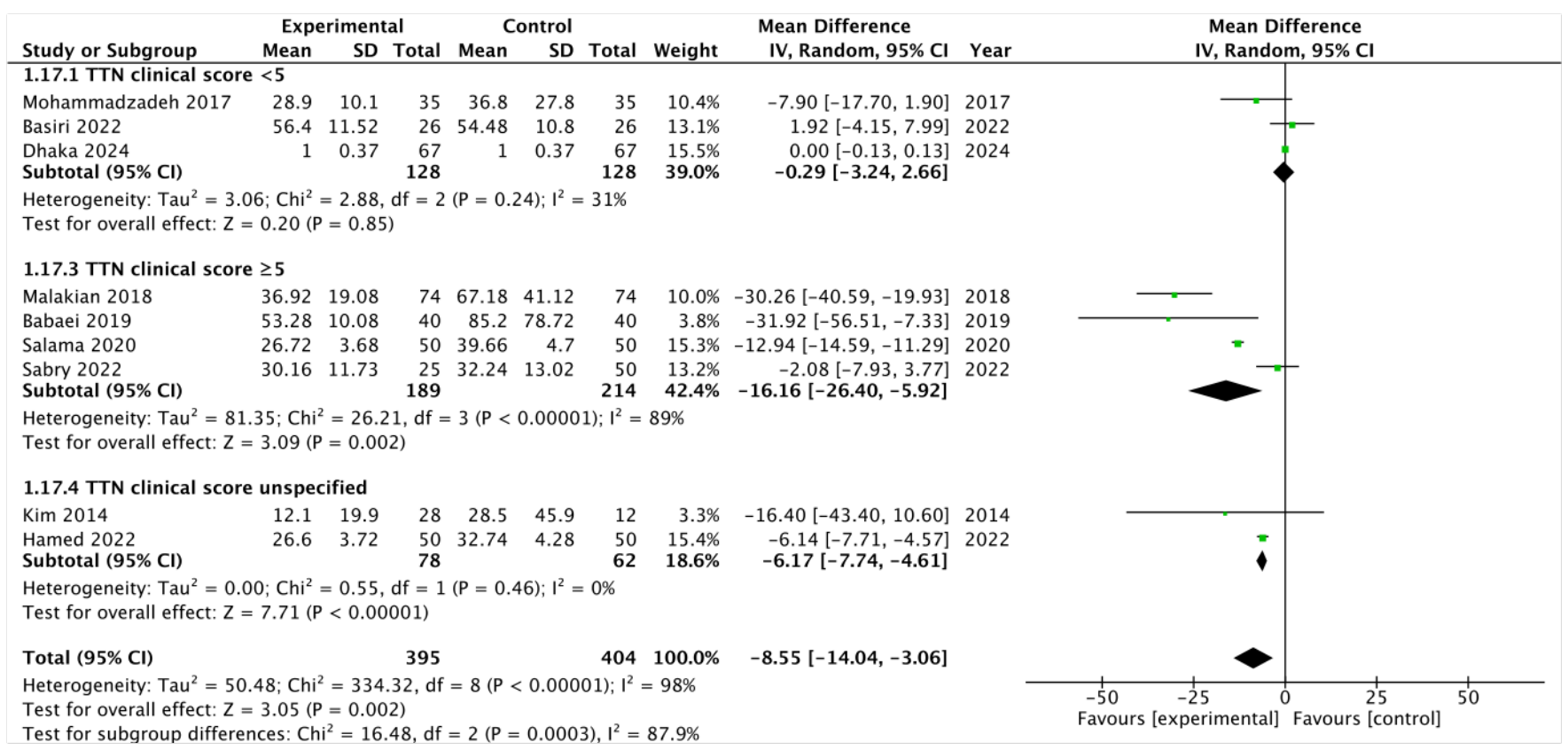

(C) Duration of hospitalization (days)

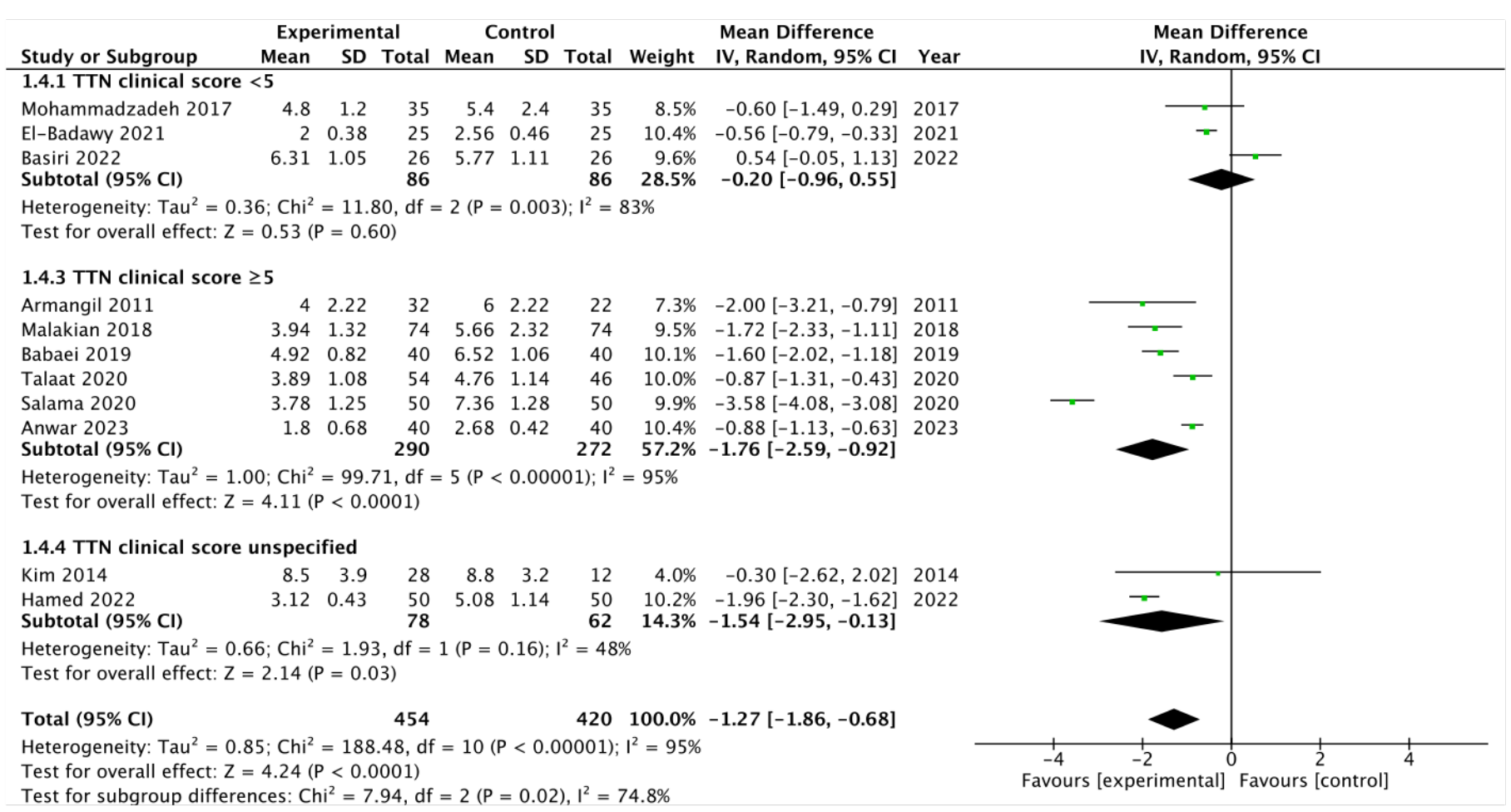

Supplement: Supplementary file 7 [file pone.0359663.s007.pdf]
